# Genome-Wide Analysis of Odorant-Binding Proteins and Chemosensory Proteins in the Bean bug *Riptortus pedestris*

**DOI:** 10.3389/fphys.2022.949607

**Published:** 2022-07-14

**Authors:** Jin-Bu Li, Mao-Zhu Yin, Wei-Chen Yao, Sai Ma, Youssef Dewer, Xing-Zhou Liu, Yue-Ying Wang, Chao-Wei Wang, Bao-Ping Li, Xiu-Yun Zhu

**Affiliations:** ^1^ Department of Entomology, College of Plant Protection, Nanjing Agricultural University, Nanjing, China; ^2^ Institute of Plant Protection, Suzhou Academy of Agricultural Sciences, Suzhou, China; ^3^ Anhui Province Key Laboratory of Pollutant Sensitive Materials and Environmental Remediation, Anhui Provincial Engineering Laboratory for Efficient Utilization of Featured Resource Plants, College of Life Sciences, Huaibei Normal University, Huaibei, China; ^4^ Phytotoxicity Research Department, Central Agricultural Pesticide Laboratory, Agricultural Research Center, Giza, Egypt

**Keywords:** *Riptortus pedestris*, genome analysis, odorant-binding proteins, chemosensory proteins, olfactory

## Abstract

Insects have sensitive olfactory systems to interact with environment and respond to the change in host plant conditions. Key genes in the system can be potential targets for developing new and efficient pest behaviour control methods. *Riptortus pedestris* is an important soybean pest in East Asia and has caused serious damage to the soybean plants in Huang-Huai-Hai region of China. However, the current treatment of pests is dominated by chemical insecticides and lacks efficient sustainable prevention and control technologies. In this study, we identified 49 putative odorant-binding proteins (OBPs) (43 were new genes) and 25 chemosensory proteins (CSPs) (17 were new genes) in *R. pedestris* genome. These OBP and CSP genes are clustered in highly conserved groups from other hemipteran species in phylogenetic trees. Most *RpedOBPs* displayed antennal-biased expression. Among the 49 *RpedOBPs*, 33 were significantly highly expressed in the antennae, including three male-biased and nine female-biased. While many *RpedCSPs* were detected both in the antennae and in non-antennal tissues, only 11 *RpedCSPs* displayed antennal-biased expression, in which four *RpedCSPs* were male-biased and five *RpedCSPs* were female-biased. Some *OBP* and *CSP* genes showed sex-biased expression profiles. Our results not only provide a foundation for future exploration of the functions of RpedOBPs and RpedCSPs but also aid in developing environmentally friendly insecticides in the future.

## Introduction

Insects have a sensitive olfactory system, which enhances their ability to adapt to the complex external environment to accurately complete behavioural reactions such as feeding, mating, and avoiding natural enemies (Leal, 2013; Robertson, 2019). A large amount of studies on the molecular mechanisms of insect olfactory systems have found that the accurate operation of the system is inseparable from the participation of olfactory genes such as odorant-binding proteins (OBPs), chemosensory proteins (CSPs), and olfactory receptors (ORs) (Zhang et al., 2013; Glaser et al., 2015; Li et al., 2015; Elfekih et al., 2016; Larter et al., 2016; Paula et al., 2016; Renou and Anton, 2020; Rihani et al., 2021).

OBPs and CSPs are located in the lymph of insect antennal sensilla, and can accurately bind to external odorants and transport them to the corresponding ORs, ionotropic receptors (IRs) or gustatory receptors (GRs) to initiate behavioural responses. Therefore, the action of OBPs and CSPs is the first step in activating insect olfactory perception (Zhou, 2010; Dani et al., 2011; Pelosi et al., 2018; Rihani et al., 2021), which can be used as potential target genes to develop new and efficient pest behaviour control agents. Since the discovery of the first OBP and CSP in *Antheraea polyphemus* (Vogt and Riddiford, 1981) and *Drosophila melanogaster* (McKenna et al., 1994), respectively, a large number of OBPs and CSPs have been confirmed and studied in different insects (Latorre-Estivalis et al., 2021; Li et al., 2021; He et al., 2022).

These two types of genes have been the subject of studies on evolution, molecular structure, tissue distribution, and functional analysis (Spinelli et al., 2012; Pelosi et al., 2018; Li et al., 2021). It is now clear that both OBPs and CSPs are soluble small-molecule proteins. Generally, OBPs use six positionally conserved cysteines to form three interlocking disulfide bridges that stabilise the three-dimensional structure of the proteins. OBPs can be divided into three distinct subfamilies: classic OBPs (six conserved cysteines), minus-C OBPs (four conserved cysteines), and plus-C OBPs (more than six conserved cysteines) (Zhou, 2010; Schultze et al., 2012; Spinelli et al., 2012; Li et al., 2013; He and He, 2014; Zhang et al., 2017b). Compared with OBPs, CSPs are smaller, display greater evolutionary conservation, and have only two disulfide bridges with four conserved cysteines (Maleszka and Stange, 1997; Bohbot et al., 1998; Pelosi et al., 2005; Zhang et al., 2014; Zhu J. et al., 2016; Yi et al., 2017; Pelosi et al., 2018). Additionally, OBPs are often specifically or highly expressed in the antennae and are mainly involved in odorant recognition (Krieger et al., 1996; Sengul and Tu, 2010; Missbach et al., 2015; Zhang et al., 2017a), whereas many CSPs are expressed in the antennal and other non-olfactory organs (Pelosi et al., 2005; Vogt, 2005; Zhang et al., 2013; Zhang et al., 2014; Zhang L.-W. et al., 2016; Chen G.-L. et al., 2018). This suggests that CSPs may play both olfactory and non-olfactory roles in insects.

The bean bug *Riptortus pedestris* (Fabricius) (Hemiptera: Alydidae) is an important soybean pest in East Asia (Xu et al., 2021) and has a wide range of hosts. In addition to soybean, it can also harm Cruciferae, Gramineae, and other crops (Huang et al., 2021). *R. pedestris* damages soybeans by sucking, which results in poor growth and development of plants and insufficient pods (Chen J. H. et al., 2018). In recent years, *R. pedestris* has caused serious damage to the soybean plants in Huang-Huai-Hai region of China and has greatly reduced the yield of soybean, or lost the harvest. It has now become an important pest in China’s summer soybean producing areas (Chen J. H. et al., 2018; Li et al., 2019). However, the current treatment of pests is still dominated by the use of chemical insecticides and lacks efficient green prevention and control technologies. This has become a growing consensus that the development of green and efficient behaviour disruptors is a popular research direction based on the exploration of insect olfactory systems (Sun et al., 2011; Cui and Zhu, 2016; Qin et al., 2020). In this study, we identified 49 OBPs and 25 CSPs in the *R. pedestris* genome and found that these genes were clustered in highly conserved groups comprising OBP and CSP genes from other hemipteran species, respectively. The gene expression profiles of OBPs and CSPs showed that most RpedOBPs displayed antennal-biased expression, while many RpedCSPs were highly expressed in the antennae and non-antennal tissues, and some genes showed sex-biased expression. These results will help us identify the functions of RpedOBPs and RpedCSPs and develop environmentally friendly insecticides against this pest in the future.

## Materials and Methods

### Insect Rearing and Tissue Extraction

*R. pedestris* were fed with bean sprouts and maintained at a temperature of 26 ± 1°C under a 14:10 h light:dark photoperiod. Female and male adults, as well as larvae, were placed in insect cages for reproduction. Fifth instar larvae were collected and raised separately to obtain three-day-old virgin adults. The heads, thoraxes, abdomens, legs, wings, and antennae of virgin adults were collected. All collected samples were immediately frozen in liquid nitrogen and stored at ˗80°C for future use.

### Sequence Data Collection and Analyses

Genome data, gene, protein, RNA and annotation files of *R. pedestris* were obtained from the Genome Warehouse (https://ngdc.cncb.ac.cn/gwh/Assembly/18849/show). We used the protein data of *R. pedestris* and blasted with different database of Nr (Non-Redundant Protein Sequence), Nt (nucleotide sequence database), UniProt (The Universal Protein Resource), KOG (Clusters of orthologous groups for eukaryotic complete genomes), eggNOG (evolutionary genealogy of genes: No-supervised Orthologous Groups), Interpro (the integrative protein signature), GO (Gene Ontology), and KEGG (Kyoto Encyclopedia of Genes and Genomes) databases (Huang et al., 2021), so *R. pedestris* proteins were annotated based on homology (These data were derived from a previous genome paper). We acquired the genes and ORF sequences of OBPs and CSPs by corresponding protein ID using *R. pedestris* genomic database. To ensure the accuracy of gene sequences, we used OBP and CSP genes to blast with Nr (Non-Redundant Protein Sequence) database in NCBI BLAST (http://blast.ncbi.nlm.nih.gov/). We also selected some genes (RpedOBP8, RpedOBP37, RpedCSP5) to clone and sent to sequencing to verify the correctness of the sequences. A total of 49 OBPs and 25 CSPs were obtained based on the similarity analysis. Putative N-terminal signal peptides of all OBPs and CSPs were predicted using SignalP 4.1 (http://www.cbs.dtu.dk/services/SignalP/), (Petersen et al., 2011).

### RNA Isolation and cDNA Synthesis

Total RNA was extracted using a FastPure^®^ Cell/Tissue Total RNA Isolation Kit (Vazyme, Nanjing, China) following the manufacturer’s instructions, and RNA quality was checked using a spectrophotometre (NanoDrop^TM^ 2000; Thermo Fisher Scientific, United States). Single-stranded cDNA templates were synthesised from 1 μg of total RNA from various tissue samples using the MonScript™ RTlll Super Mix with dsDNase (Two-Step) (Monad, Shanghai, China).

### Quantitative Real Time-Polymerase Chain Reaction

The qRT-PCR primers for 49 OBPs and 25 CSPs (Supplementary Table S1) were designed using Beacon Designer 7.9 (PREMIER Biosoft International, CA, United States). Expression profilings of RpedOBPs and RpedCSPs were performed using qRT-PCR in a LightCycler^®^ 96 (Roche, Switzerland) with a mixture of 5 μL MonAmp™ ChemoHS qPCR Mix (Monad, Shanghai, China), 0.2 μL of each primer (10 μM), 2.5 ng of sample cDNA, and 3.6 μL of sterilised ultrapure H_2_O. The reaction program was as follows: 10 min at 95°C, 40 cycles at 95°C for 10 s, 60°C for 10 s, and 72°C for 30 s. The results were analysed using LightCycler® 96 SW 1.1. Then, fluorescence was measured over a 55–95°C melting curve to detect a single gene-specific peak and to check the absence of primer dimer peaks; a single and discrete peak was detected for all primers tested. Negative controls consisted of non-template reactions in which the cDNA was replaced with H_2_O.

The expression levels of RpedOBPs and RpedCSPs were calculated relative to the reference genes *RpedGAPDH* (*R. pedestris* glyceraldehyde-3-phosphate dehydrogenase) and *RpedEF* (*R. pedestris* elongation factor) using the Q-Gene method in Microsoft Excel-based software Visual Basic (Muller et al., 2002; Simon, 2003). For each sample, three biological replicates were performed with three technical replicates per biological replicate.

### Sequence Analyses

Based on sequence alignments, all phylogenetic trees in this study were constructed using the MEGA7 software (Kumar et al., 2016) using the neighbour-joining method, and each tree was tested by bootstrapping with 1,000 replicates. A phylogenetic tree was constructed based on the alignment results of cytochrome oxidase subunit I (COI) genes from different species (*Aphis gossypii*: KR017753.1, *Nilaparvata lugens*: AB325705.1, *Sogatella furcifera*: LC005703.1, *Adelphocoris lineolatus*: MZ608737.1, *Adelphocoris suturalis*: KY367052.1, *Nysius ericae*: KM022105.1, and *R. pedestris*: MG838422.1). The amino acid sequences of the RpedOBPs, RpedCSPs, and other hemipteran OBPs and CSPs were listed in Supplementary Table S2. The totals numbers of OBPs and CSPs in other insects have been reported in previous studies (Gu et al., 2011; Gu et al., 2013; Cao et al., 2014; He and He, 2014; Xue et al., 2014; Yang et al., 2014; Zhou et al., 2014; He et al., 2015; Zhou et al., 2015; Cui et al., 2017). Gene structure and exon/intron structure maps were generated using TBTools (version 1.098728) (Chen et al., 2020) based on *R. pedestris* annotated file (Gene Location Visualisation from GTF/GFF and Visualisation of Gene Structure). Pairwise similarity of sequences was also generated by TBTools based protein sequences (Protein Pairwise Similarity Matrix). Expression heat maps and bars were drawn using TBtools and GraphPad Prism 9.0, respectively, based on the qRT-PCR results.

### Statistical Analysis

The qRT-PCR data (mean ± SE) of RpedOBPs and RpedCSPs from various samples were subjected to one-way nested analysis of variance (ANOVA) followed by a least significant difference test (LSD) to compare means using the SPSS Statistics software (version 22.0; SPSS Inc., Chicago, IL, United States).

## Results and Discussion

### Identification of OBP and CSP Genes in *R. pedestris*

Recent progress in the whole-genome sequencing provides insights into the molecular mechanisms of olfaction in insects (Cheng et al., 2017; Wan et al., 2019). Second- and third-generation sequencing methods have also been successfully used for *R. pedestris* genome assembly. Based on BUSCO completeness and contig length, the Wtdbg2 assembly was used for the draft assembly of the *R. pedestris* genome (Huang et al., 2021). We downloaded the *R. pedestris* genome, protein, RNA and annotation files using Genome Warehouse and further annotated them using the Nr, Nt, SwissProt, KOG, eggNOG, Interpro, GO, and KEGG databases. A total of 53 OBPs and 25 CSPs of *R. pedestris* were identified and corrected using the following correction through BLAST. Finally, 49 OBPs and 25 CSPs were identified and named RpedOBP1-49 (43 were new genes), RpedCSP1-25 (17 were new genes) (Table 1). The predicted results of the sequences analysis revealed that all OBPs and CSPs had full-length open reading frames (ORF), and 39 OBPs and 23 CSPs had a signal peptide, respectively. The 49 OBPs without signal peptides share 22.5–99.19% amino acid identities with each other, while the 25 CSPs share 22.5–99.07% amino acid identities with each other (Supplementary Table S3). Full-length sequences of the 49 RpedOBPs and 25 RpedCSPs were presented in the supplementary files.

**TABLE 1:** The sequences information of RpedOBPs and RpedCSPs.

Gene	Gene	ORF	Signal	Complete	Best blastx match					
Name	ID	(aa)	Peptide	ORF	Name	Acc. number	Subject ORF(aa)	Species	E value	Identity (%)
Odorant binding protein (OBP)										
RpedOBP1	Rp.chr1.1194	170	N	Y	odorant-binding protein 1	AWW17235.1	223	*Riptortus pedestris*	6E-10	43
RpedOBP2	Rp.chr1.1641	331	N	Y	odorant-binding protein 5	AOV87022.1	224	*Halyomorpha halys*	1E-48	41
RpedOBP3	Rp.chr1.1971	173	1–16	Y	general odorant-binding protein 70	XP_014286615.1	225	*Halyomorpha halys*	2E-83	80
RpedOBP4	Rp.chr2.0376	170	1–19	Y	odorant-binding protein 7	AYN07348.1	226	*Yemma signatus*	1E-74	71
RpedOBP5	Rp.chr2.0393	215	1–25	Y	odorant-binding protein 19	AXB87334.1	227	*Tropidothorax elegans*	5E-24	34
RpedOBP6	Rp.chr2.0394	280	1–21	Y	odorant-binding protein 11	AYN07352.1	228	*Yemma signatus*	2E-27	46
RpedOBP7*	Rp.chr2.0395	215	N	Y	odorant-binding protein 2	AWW17236.1	229	*Riptortus pedestris*	2E-129	100
RpedOBP8*	Rp.chr2.0396	223	1–22	Y	odorant-binding protein 1	AWW17235.1	230	*Riptortus pedestris*	3E-126	99
RpedOBP9	Rp.chr2.0983	248	N	Y	odorant binding protein 47	QCZ25104.1	231	*Nezara viridula*	5E-86	58
RpedOBP10	Rp.chr2.1234	165	1–24	Y	odorant binding protein 39	QCZ25096.1	232	*Nezara viridula*	9E-39	44
RpedOBP11	Rp.chr2.1239	146	1–18	Y	odorant-binding protein 15	AXB87330.1	233	*Tropidothorax elegans*	1E-53	53
RpedOBP12*	Rp.chr2.1378	153	1–18	Y	odorant-binding protein 5	AWW17239.1	234	*Riptortus pedestris*	3E-95	100
RpedOBP13	Rp.chr2.1485	211	1–20	Y	odorant-binding protein 1	AXB87316.1	235	*Tropidothorax elegans*	2E-72	73
RpedOBP14	Rp.chr2.1635	148	1–20	Y	odorant-binding protein 10	AYN07351.1	236	*Yemma signatus*	1E-40	56
RpedOBP15	Rp.chr2.1704	147	1–21	Y	odorant binding protein 45	QCZ25102.1	237	*Nezara viridula*	3E-56	67
RpedOBP16	Rp.chr2.1706	137	1–18	Y	odorant binding protein 42	QCZ25099.1	238	*Nezara viridula*	6E-7	34
RpedOBP17	Rp.chr2.2024	151	1–20	Y	odorant-binding protein 14	AXB87329.1	239	*Tropidothorax elegans*	1E-67	74
RpedOBP18*	Rp.chr2.2170	142	1–18	Y	odorant-binding protein 6	AWW17240.1	240	*Riptortus pedestris*	3E-100	100
RpedOBP19	Rp.chr2.3105	159	1–24	Y	odorant-binding protein 4	AWW17238.1	241	*Riptortus pedestris*	8E-89	91
RpedOBP20	Rp.chr2.3220	144	1–21	Y	odorant-binding protein 11	AXB87326.1	242	*Tropidothorax elegans*	1E-21	38
RpedOBP21	Rp.chr2.3271	148	1–27	Y	odorant binding protein 14	QCZ25071.1	243	*Nezara viridula*	3E-53	65
RpedOBP22	Rp.chr2.3272	136	1–17	Y	odorant-binding protein 4	AYN07346.1	244	*Yemma signatus*	2E-52	71
RpedOBP23	Rp.chr2.3273	109	N	Y	odorant binding protein 34	QCZ25091.1	245	*Nezara viridula*	5E-42	61
RpedOBP24	Rp.chr2.3274	155	1–19	Y	odorant-binding protein 3	AXB87318.1	246	*Tropidothorax elegans*	1E-34	51
RpedOBP25	Rp.chr2.3275	151	1–17	Y	odorant binding protein 41	QCZ25098.1	247	*Nezara viridula*	3E-51	65
RpedOBP26	Rp.chr2.3276	121	N	Y	odorant binding protein 41	QCZ25098.1	248	*Nezara viridula*	8E-14	40
RpedOBP27	Rp.chr2.3278	149	1–17	Y	odorant binding protein 41	QCZ25098.1	249	*Nezara viridula*	2E-12	31
RpedOBP28	Rp.chr2.3320	309	1–17	Y	odorant binding protein 41	QCZ25098.1	250	*Nezara viridula*	5E-12	36
RpedOBP29	Rp.chr2.3321	148	1–22	Y	odorant-binding protein 11	AXB87326.1	251	*Tropidothorax elegans*	1E-18	43
RpedOBP30	Rp.chr2.3326	148	1–22	Y	odorant-binding protein 11	AXB87326.1	252	*Tropidothorax elegans*	3E-13	36
RpedOBP31	Rp.chr2.3445	94	N	Y	odorant binding protein 16	QCZ25073.1	253	*Nezara viridula*	1E-8	32
RpedOBP32	Rp.chr2.3446	184	N	Y	odorant binding protein 15	QCZ25072.1	254	*Nezara viridula*	2E-10	33
RpedOBP33	Rp.chr2.3447	129	1–16	Y	odorant binding protein 42	QCZ25099.1	255	*Nezara viridula*	3E-11	39
RpedOBP34	Rp.chr3.2341	149	N	Y	odorant-binding protein 10	AXB87325.1	256	*Tropidothorax elegans*	2E-41	48
RpedOBP35	Rp.chr3.2343	146	1–19	Y	odorant-binding protein 10	AXB87325.1	257	*Tropidothorax elegans*	1E-39	52
RpedOBP36	Rp.chr3.2431	146	1–21	Y	odorant-binding protein 14	AYN07355.1	258	*Yemma signatus*	3E-31	44
RpedOBP37	Rp.chr3.2644	132	1–18	Y	odorant-binding protein 1	AYN07343.1	259	*Yemma signatus*	1E-61	77
RpedOBP38*	Rp.chr5.1134	153	1–19	Y	odorant-binding protein 7	AWW17241.1	260	*Riptortus pedestris*	2E-108	100
RpedOBP39*	Rp.chr5.2242	147	1–23	Y	odorant-binding protein 3	AWW17237.1	261	*Riptortus pedestris*	5E-49	100
RpedOBP40	Rp.chr5.2243	144	1–20	Y	odorant-binding protein 3	AWW17237.1	262	*Riptortus pedestris*	1E-47	98
RpedOBP41	Rp.chr5.2244	146	1–20	Y	odorant-binding protein 10	AXB87325.1	263	*Tropidothorax elegans*	4E-36	47
RpedOBP42	Rp.chr5.2493	196	1–23	Y	odorant binding protein 24	QCZ25081.1	264	*Nezara viridula*	4E-10	28
RpedOBP43	Rp.chr5.2533	134	1–19	Y	general odorant-binding protein 56 h	XP_016995790.1	265	*Drosophila takahashii*	3E-6	27
RpedOBP44	Rp.chr5.2536	148	1–21	Y	odorant binding protein 3	KAF2903755.1	266	*Sirex nitobei*	3E-4	36
RpedOBP45	Rp.chr5.2537	152	1–21	Y	odorant-binding protein 2	AIX97125.1	267	*Rhyzopertha dominica*	9E-6	41
RpedOBP46	Rp.chr5.2538	152	1–21	Y	general odorant-binding protein 19d-like	XP_031341271.1	268	*Photinus pyralis*	3E-7	35
RpedOBP47	Rp.chr5.2539	144	1–19	Y	odorant binding protein 13	QCZ25070.1	269	*Nezara viridula*	0.02	29
RpedOBP48	Rp.chr5.2578	141	1–22	Y	odorant binding protein 56	QCZ25112.1	270	*Nezara viridula*	7E-12	30
RpedOBP49	Rp.scaffold.288	125	N	Y	odorant binding protein 56	QCZ25112.1	271	*Nezara viridula*	8E-11	28
Chemosensory Protein (CSP)										
RpedCSP1	Rp.chr1.2826	120	1–16	Y	chemosensory protein	AID61322.1	121	*Calliphora stygia*	2E-26	43
RpedCSP2	Rp.chr3.0416	128	N	Y	putative chemosensory protein	SAJ59007.1	113	*Triatoma brasiliensis*	3E-50	75
RpedCSP3*	Rp.chr3.2183	126	1–16	Y	chemosensory protein 7	AWW17231.1	126	*Riptortus pedestris*	1E-56	100
RpedCSP4*	Rp.chr3.2650	133	1–20	Y	chemosensory protein 5	AWW17229.1	133	*Riptortus pedestris*	2E-65	100
RpedCSP5*	Rp.chr3.2651	131	1–19	Y	chemosensory protein 10	AWW17234.1	131	*Riptortus pedestris*	3E-74	100
RpedCSP6	Rp.chr3.2661	127	1–19	Y	chemosensory protein 1	AWW17225.1	127	*Riptortus pedestris*	7E-72	84
RpedCSP7	Rp.chr3.2662	127	1–19	Y	chemosensory protein 1	AWW17225.1	127	*Riptortus pedestris*	7E-72	84
RpedCSP8	Rp.chr3.2682	134	1–15	Y	chemosensory protein	AVM86426.1	131	*Corythucha ciliata*	3E-33	45
RpedCSP9	Rp.chr3.2683	126	1–16	Y	chemosensory protein 7	AWW17231.1	126	*Riptortus pedestris*	4E-45	68
RpedCSP10	Rp.chr3.2684	127	1–16	Y	chemosensory protein 5	QCZ25119.1	126	*Nezara viridula*	3E-51	70
RpedCSP11	Rp.chr3.2687	139	1–16	Y	chemosensory protein CSP3	ABM67690.1	126	*Spodoptera exigua*	2E-22	39
RpedCSP12*	Rp.chr3.2688	133	1–18	Y	chemosensory protein 3	AWW17227.1	133	*Riptortus pedestris*	5E-76	100
RpedCSP13	Rp.chr3.2689	187	1–16	Y	chemosensory protein 7	AWW17231.1	126	*Riptortus pedestris*	4E-29	63
RpedCSP14	Rp.chr3.2690	127	1–16	Y	chemosensory protein 1	AWW17225.1	127	*Riptortus pedestris*	3E-64	83
RpedCSP15	Rp.chr3.2692	127	1–16	Y	chemosensory protein 1	AWW17225.1	127	*Riptortus pedestris*	1E-63	82
RpedCSP16*	Rp.chr3.2693	127	1–19	Y	chemosensory protein 1	AWW17225.1	127	*Riptortus pedestris*	6E-75	100
RpedCSP17	Rp.chr3.2694	127	1–19	Y	chemosensory protein 1	AWW17225.1	127	*Riptortus pedestris*	5E-68	91
RpedCSP18	Rp.chr3.2695	127	1–19	Y	chemosensory protein 1	AWW17225.1	127	*Riptortus pedestris*	2E-67	91
RpedCSP19	Rp.chr3.2696	127	1–19	Y	chemosensory protein 1	AWW17225.1	127	*Riptortus pedestris*	2E-68	92
RpedCSP20	Rp.chr3.2697	129	1–16	Y	chemosensory protein 4	AWW17228.1	121	*Riptortus pedestris*	1E-63	91
RpedCSP21	Rp.chr3.2698	130	1–16	Y	chemosensory protein 4	AWW17228.1	121	*Riptortus pedestris*	6E-68	98
RpedCSP22*	Rp.chr3.2699	132	1–16	Y	chemosensory protein 9	AWW17233.1	132	*Riptortus pedestris*	7E-79	100
RpedCSP23*	Rp.chr5.2503	125	1–17	Y	chemosensory protein 2	AWW17226.1	125	*Riptortus pedestris*	5E-87	100
RpedCSP24*	Rp.chrX.0381	155	N	Y	chemosensory protein 6	AWW17230.1	130	*Riptortus pedestris*	3E-54	99
RpedCSP25	Rp.chrX.0979	121	1–16	Y	chemosensory protein 7	QCZ25121.1	124	*Nezara viridula*	3E-50	70

*indicates that this gene has been saved in GenBank by other researchers.

**FIGURE 1:**
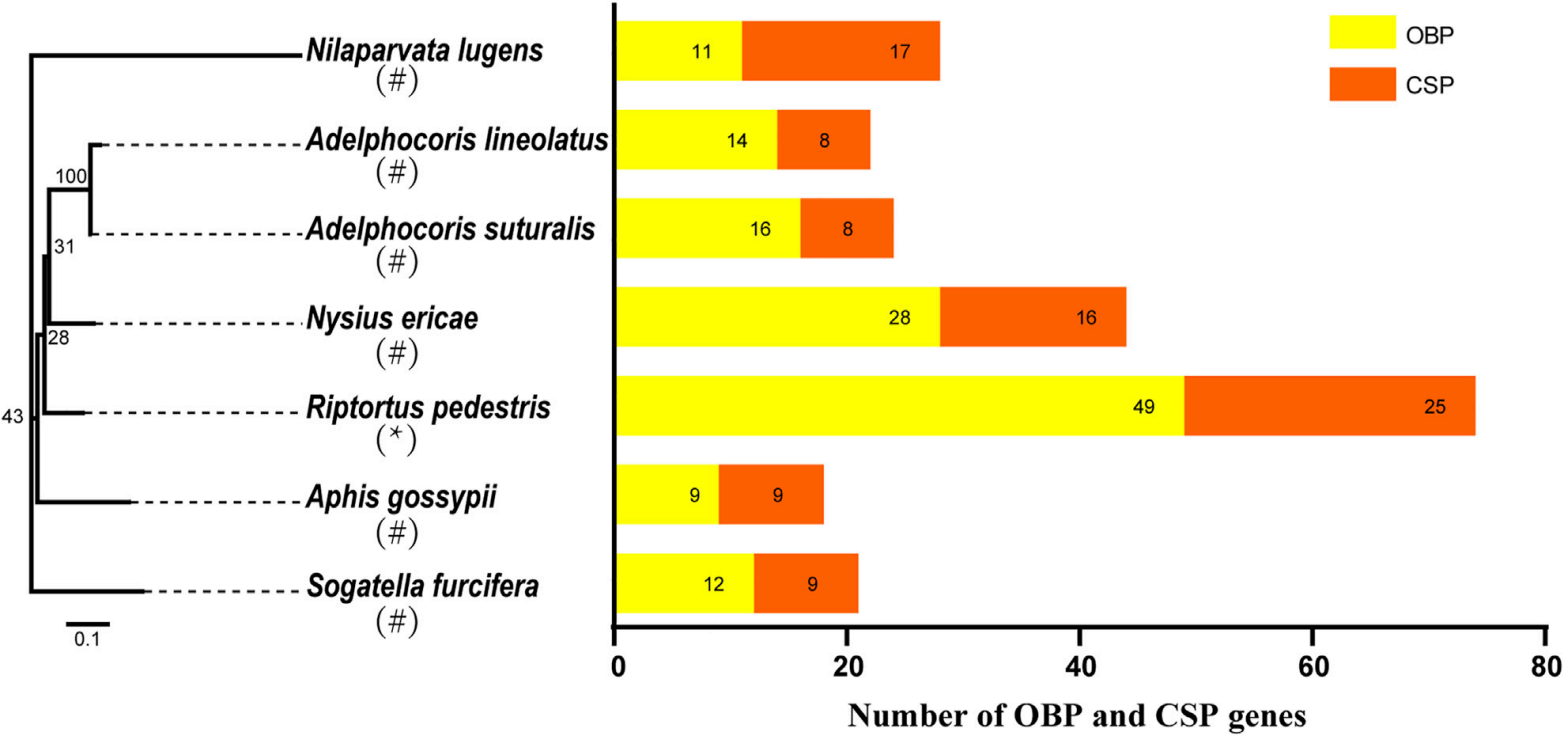
The number of OBP and CSP genes in different insect species, obtained from genome (*) or antennal transcriptome (#). The digits by the histogram bars represent number of OBP and CSP genes in different hemipteran species (*Aphis gossypii*, *Nilaparvata lugens*, *Sogatella furcifera*, *Adelphocoris lineolatus*, *Adelphocoris suturalis*, *Nysius ericae*, *R. pedestris*) and phylogenetic tree was built by these hemipteran species COI genes.

The number of RpedOBP and RpedCSP genes identified for *R. pedestris* is larger than those in other hemipterans (Figure 1). For example, 11 OBPs and 17 CSPs were identified in *N. lugens* (Xue et al., 2014; Yang et al., 2014; Zhang Y.-N. et al., 2016), 14 OBPs and 8 CSPs in *A. lineolatus* (Gu et al., 2011; Zhang Y.-N. et al., 2016), 16 OBPs and eight CSPs in *A. suturalis* (Cui et al., 2017), 28 OBPs and 16 CSPs in *N. ericae* (Zhang Y.-N. et al., 2016), nine OBPs and nine CSPs in *A. gossypii* (Gu et al., 2013; Zhang Y.-N. et al., 2016), and 12 OBPs and nine CSPs were identified in *S. furcifera* (He and He, 2014; Zhou et al., 2015). The differences in gene numbers may be explained by: 1) the different behaviours of different insects requiring distinct molecular mechanisms that have been developed over evolutionary time, and 2) the genomic data of *R. pedestris* that is more conducive to the comprehensive mining of OBP and CSP genes than other hemipteran species.

### Localization of OBPs and CSPs in the *R. pedestris* Genome

**FIGURE 2:**
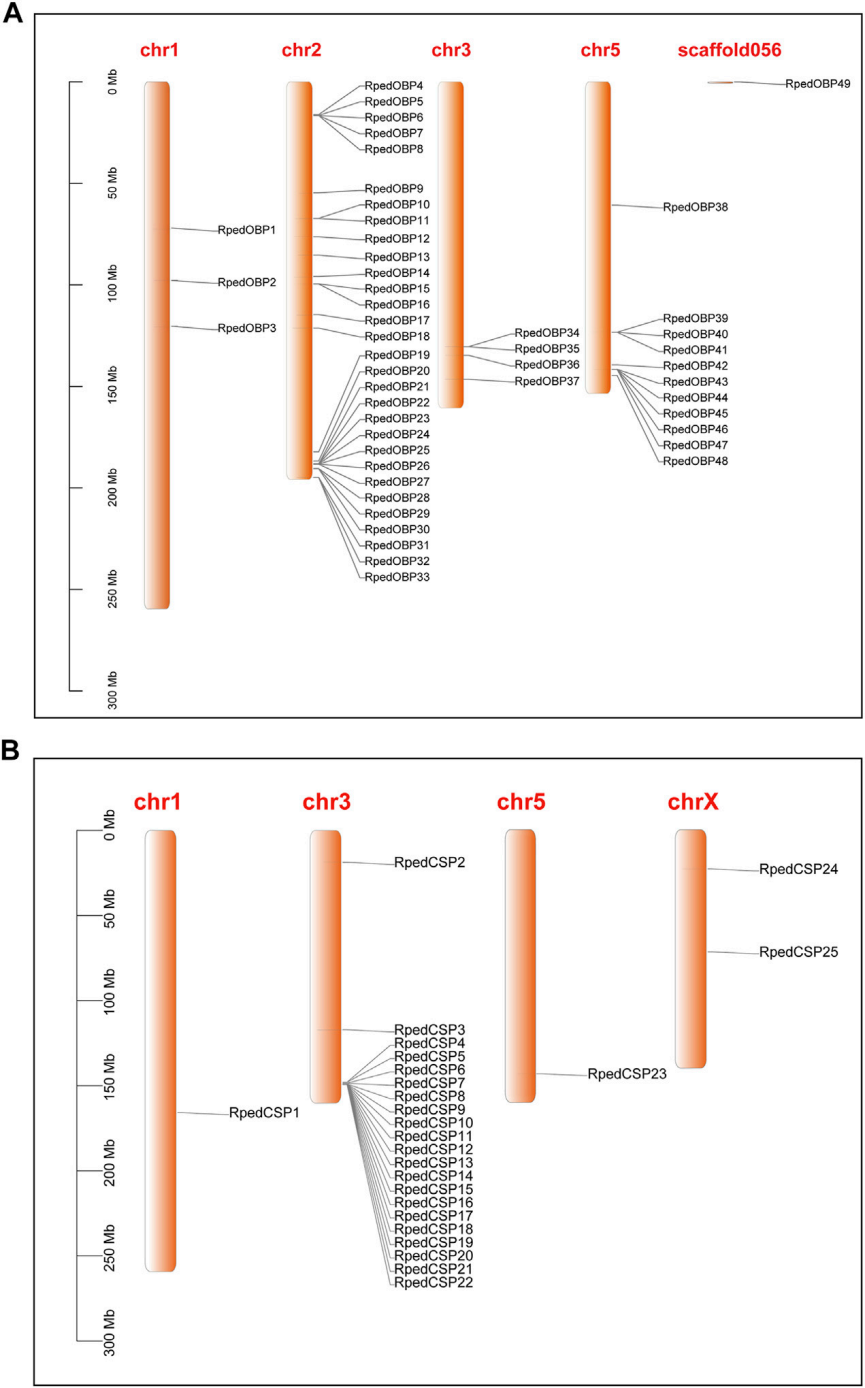
Localization of OBPs and CSPs in the *R. pedestris* genome. Based on the annotation file of *R. pedestris*, we acquired the localization of OBP and CSP genes in genome. **(A)** The *RpedOBPs* in the *R. pedestris* genome, they clustered together in 4 chromosomes and one scaffold. **(B)** The *RpedCSPs* in the *R. pedestris* genome, they clustered together in 4 chromosomes.

To clarify the position of OBPs and CSPs in chromosomes, we carried out chromosome location analysis of all genes, and the results showed that 48 OBP genes were distributed across four chromosomes (Figure 2A); only *RpedOBP49* was located on scaffold056, which could not be mapped to a chromosome based on the current genome assembly. Thirty OBPs clustered together on chromosome 2, followed by chromosome 5 (11 OBPs), 3 (four OBPs), and 1 (three OBPs). Twenty-five CSP genes were distributed across four chromosomes, with 21 CSPs clustered together on chromosome 3 and the others on chromosome X (two CSPs), 1 (one CSP), and 5 (one CSP) (Figure 2B). More than 60% OBP and 80% CSP genes are located within clusters, as seen in other insects (Gong et al., 2009; Gu et al., 2013), indicating a relatively recent expansion of the OBP and CSP families of *R. pedestris* and the diverse functions of genes have evolved in response to different odorants in the environment.

### Genomic Structure of *R. pedestris* OBPs and CSPs

**FIGURE 3:**
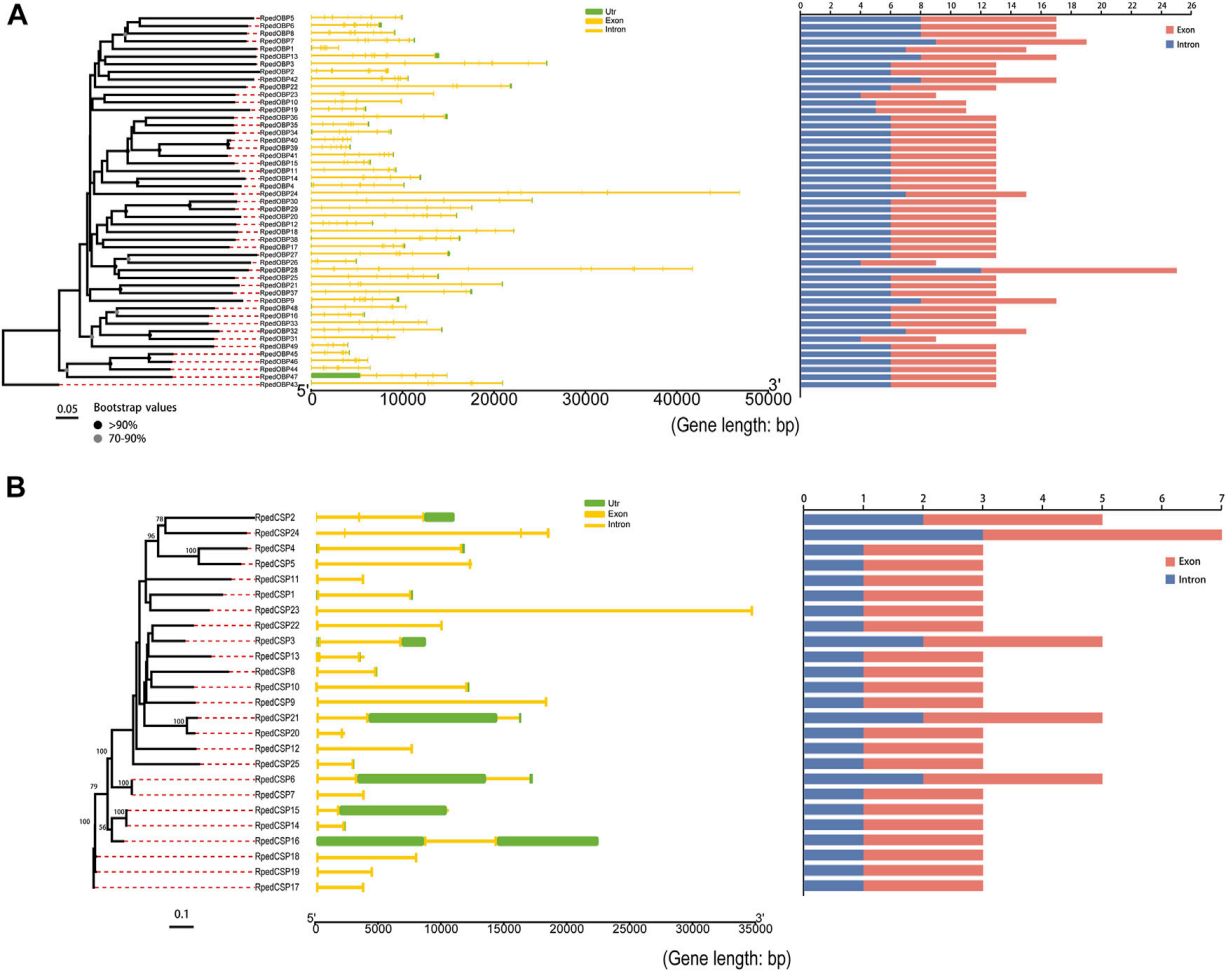
Genomic structure of *R. pedestris* OBPs and CSPs. Based on the annotation file of *R. pedestris,* we acquired genomic structures of OBP and CSP genes. **(A)** Numbers of exon/intron and exon/intron structures of *RpedOBPs.* **(B)** Numbers of exon/intron and exon/intron structures of *RpedCSPs.*

To further clarify the genomic structural characteristics of OBPs and CSPs, we obtained the gene lengths and intron numbers of OBPs and CSPs based on the genome annotation file of *R. pedestris* (Figure 3). The lengths of the OBP genes ranged from 3.065 to 46.888 kb, with 33 OBPs having six introns and the other 16 OBPs having four, five, seven, eight, nine, and 12 introns, respectively (Figure 3A). The CSP genes were much shorter, ranging from 2.114 to 34.628 kb, with one, two, or three introns (Figure 3B). The phylogenetic trees of OBPs and CSPs in *R. pedestris* showed that the genes clustered together tended to have similar genomic structures, which also implies that they may have similar functions. The sequences of RpedOBPs were longer and had more introns than those of RpedCSPs, indicating that they may have complex features of functional differentiation.

### Phylogenetic Analyses of Hemipteran OBPs and CSPs

**FIGURE 4:**
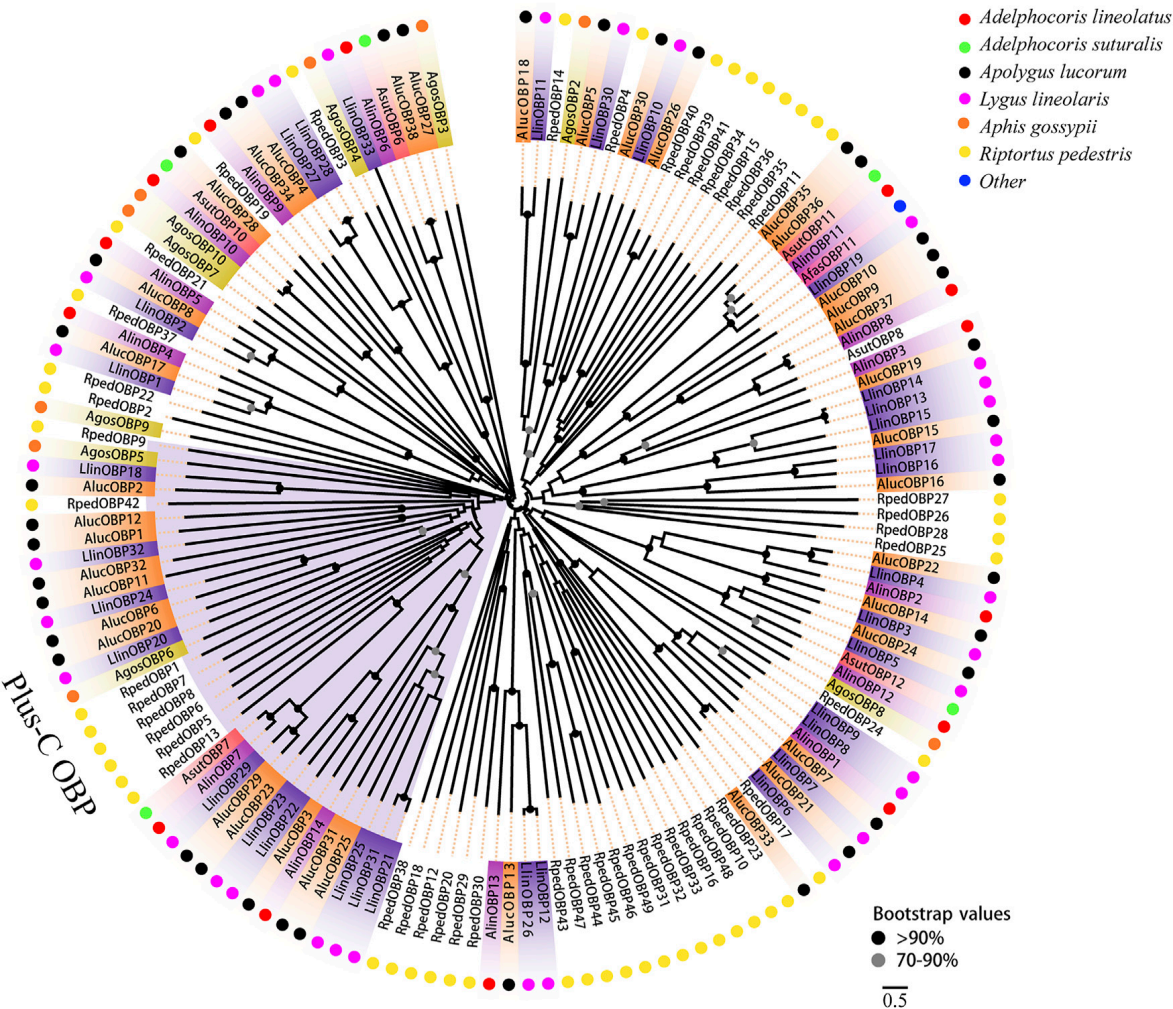
Phylogenetic tree of OBPs. Phylogenetic neighbor-joining tree was constructed using MEGA7 based on alignment results of MEGA7. A phylogenetic neighbor-joining tree was constructed using the OBPs protein that removed signal peptide. Bootstrap values higher than 70 were displayed.

**FIGURE 5:**
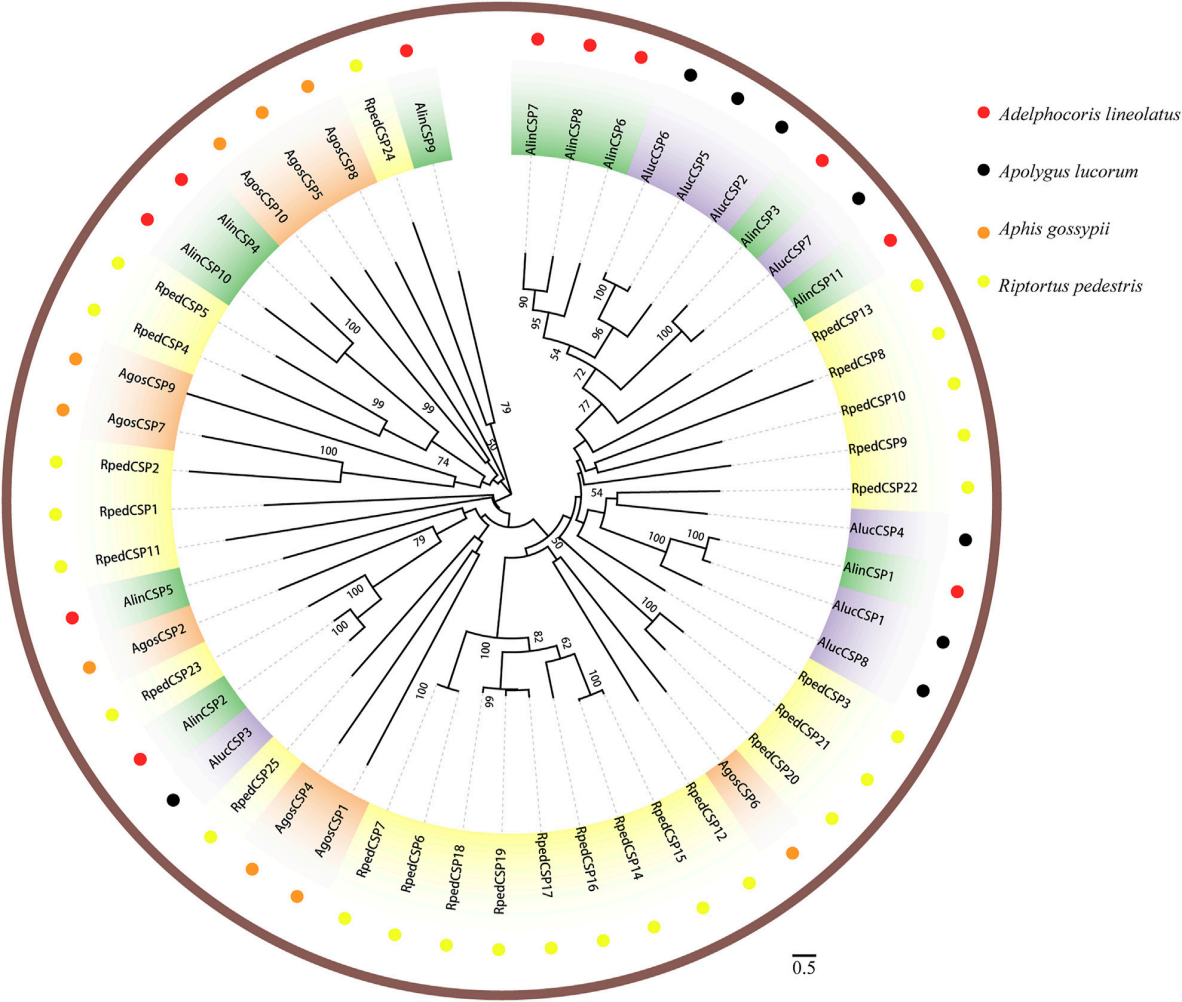
Phylogenetic tree of CSPs. Phylogenetic neighbor-joining tree was constructed using MEGA7 based on alignment results of MEGA7. A phylogenetic neighbor-joining tree was constructed using the CSPs protein that removed signal peptide. Bootstrap values higher than 50 were displayed.

Two phylogenetic trees were constructed for the OBPs and CSPs using protein sequences from *R. pedestris*, *A. lineolatus*, *Apolygus lucorum*, *Aphis gossypii*, and other hemipteran species (Gu et al., 2011; Gu et al., 2013; Cao et al., 2014; He and He, 2014; Xue et al., 2014; Yang et al., 2014; Zhou et al., 2014; He et al., 2015; Zhou et al., 2015; Cui et al., 2017). Similar to that in other studies (Gu et al., 2011; Zhang Y.-N. et al., 2016; Cui et al., 2017), the OBP tree in this study showed that eight RpedOBPs (OBP1, 5–9, 13, and 42) could be divided into the Plus-C OBP subfamily, and the other 41 RpedOBPs clustered into the classic OBP subfamily (Figure 4). In the constructed CSP tree, our results indicated that all 25 RpedCSPs were distributed along various branches, and each clustered with at least one other moth orthologue (Figure 5). The diversity of the RpedOBPs and RpedCSPs families suggests a role for positive selection in the rapid evolution and functional diversification of these genes. We speculate that both RpedOBP and RpedCSP genes had some gene expansions, such as OBP1/5/6/7/8/13, OBP23/10/48/16/33/32/31/49/46/45/44/47/43, OBP25/26/27/28, OBP40/39/41/34/15/36/35/11, CSP13/8/19/22, and CSP12/15/14/16/17/19/18/6/7, indicating that these genes may be involved in the recognition of important odorants related to *R. pedestris* behaviour (Pelosi et al., 2005; Matsuo et al., 2007; Gu et al., 2012; Poivet et al., 2013; Martin-Blazquez et al., 2017; He et al., 2019).

### Expression Profiles of *R. pedestris* OBP and CSP Genes

**FIGURE 6:**
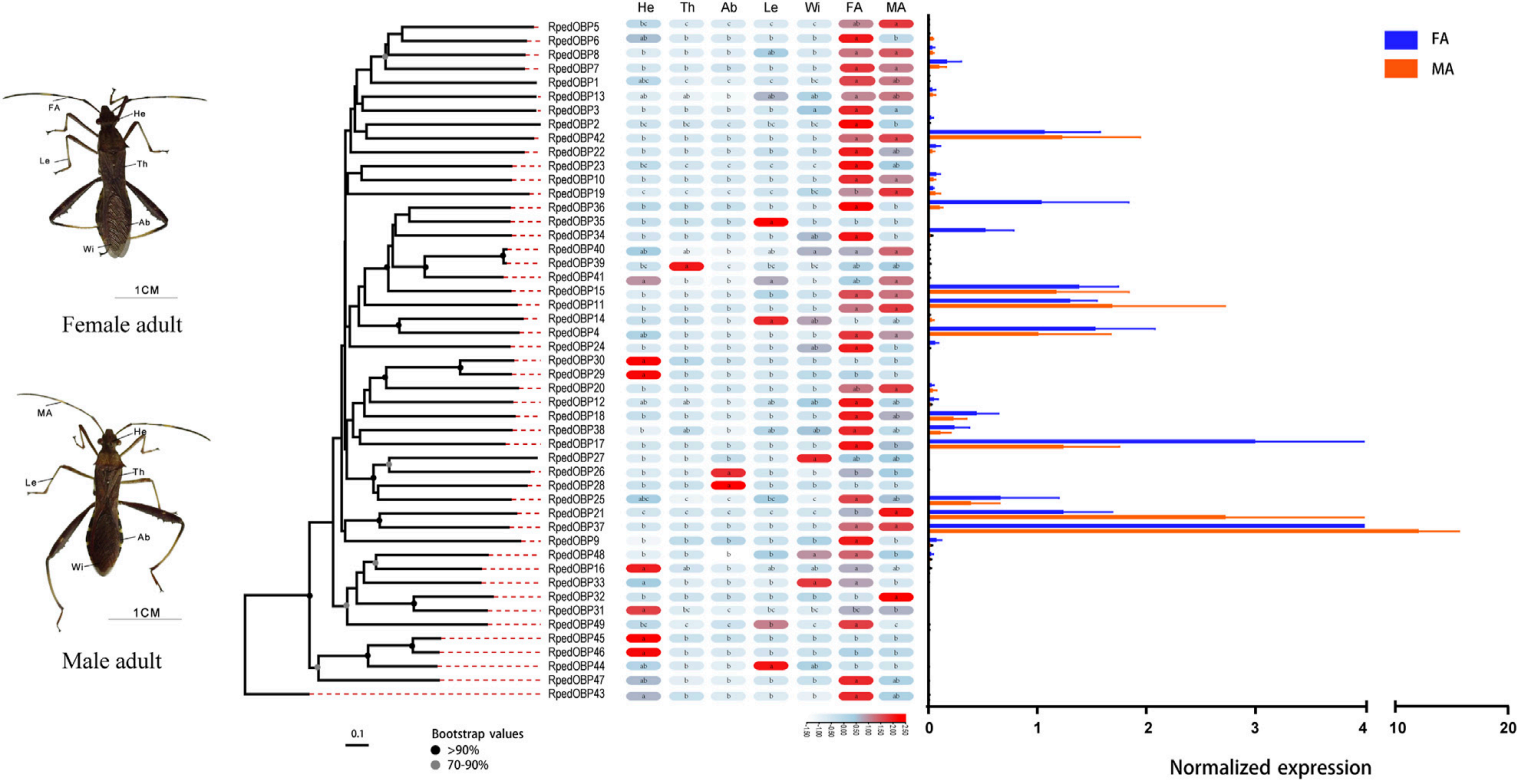
Tissue expression profiles of *R. pedestris* OBPs by qRT-PCR. The relative expression level is presented as mean ± SE (*n* = 3). The heatmap use Log2 and row scale based the relative expression level data. Different capital letters mean a significant difference between tissues (*p* < 0.05, ANOVA, LSD). He, heads; Th, thoraxes; Ab, abdomens; Le, legs; Wi, wings; FA, female antennae; MA, male antennae.

**FIGURE 7:**
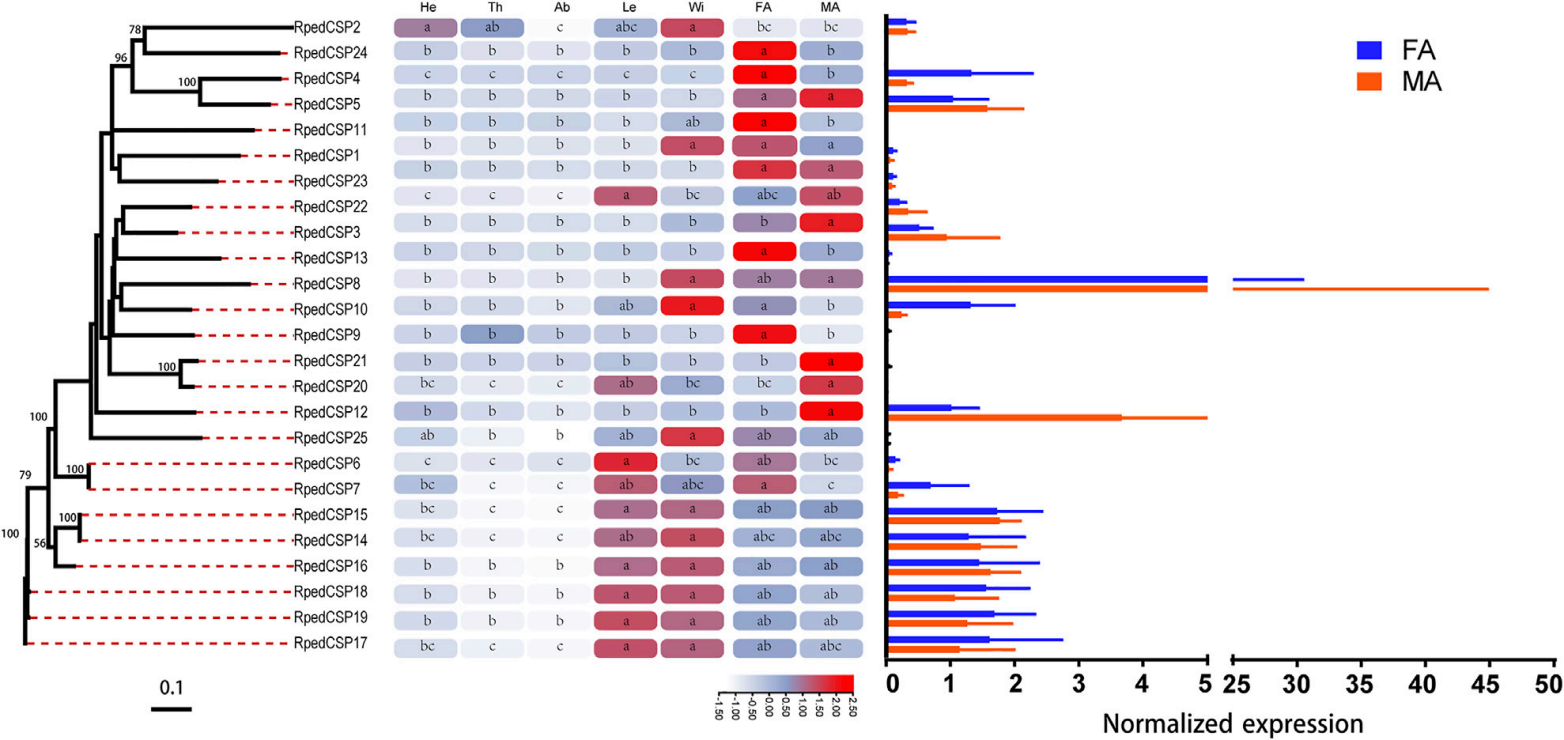
Tissue expression profiles of *R. pedestris* CSPs by qRT-PCR. The relative expression level is presented as mean ± SE (*n* = 3). The heatmap use Log2 and row scale based the relative expression level data. Different capital letters mean a significant difference between tissues (*p* < 0.05, ANOVA, LSD). He, heads; Th, thoraxes; Ab, abdomens; Le, legs; Wi, wings; FA, female antennae; MA, male antennae.

We used qRT-PCR to assess expression profiles of all *R. pedestris* OBPs and CSPs in the heads, thoraxes, abdomens, legs, wings, and antennae of the adults. The results showed that all OBPs and CSPs were expressed in the adult antennae of *R. pedestris*. Among the 49 RpedOBPs, 33 (approximately 67%) were significantly highly expressed in the antennae, including three male-biased (RpedOBP19, RpedOBP21, and RpedOBP32) and nine female-biased (RpedOBP2, RpedOBP6, RpedOBP9, RpedOBP17, RpedOBP24, RpedOBP34, RpedOBP36, RpedOBP48, and RpedOBP49). Among the 49 RpedOBPs, RpedOBP37 exhibited the highest expression level in male and female antennae (Figure 6). Compared to RpedOBPs, RpedCSPs were highly expressed in adult antennae as well as in non-antennal tissues. Of the 25 identified RpedCSP genes, only 11 RpedCSPs (approximately 44%) displayed antennal-biased expression; four RpedCSPs (RpedCSP3, RpedCSP12, RpedCSP20, and RpedCSP21) were male-biased and five RpedCSPs (RpedCSP4, RpedCSP9, RpedCSP11, RpedCSP13, and RpedCSP24) were female-biased in their expression (Figure 7). Several studies have shown that OBPs and CSPs are required for the correct recognition of some odorants from the external environment (Zhang et al., 2014; Liu et al., 2015; Chen G.-L. et al., 2018; Pelosi et al., 2018; Zhang et al., 2020a; Zhang et al., 2020b), therefore, we infer that the 33 RpedOBPs and 11 RpedCSPs highly expressed in adult antennae are likely to be involved in the crucial odorant reorganisation of *R. pedestris* (Krieger et al., 1996; Bohbot and Vogt, 2005; Zhang et al., 2014; Missbach et al., 2015; Chen G.-L. et al., 2018). The sex-biased RpedOBPs and RpedCSPs may be involved in the reorganisation of plant volatiles from oviposition sites or other sex-related odorants (He et al., 2010; Zhou et al., 2013; Zhang et al., 2019). Further analysis is needed to explore their exact roles, such as through fluorescence competitive binding assays (Liu et al., 2015; Ingham et al., 2020; Li et al., 2022), CRISPR/Cas9 mediated genome editing (Zhu G.-H. et al., 2016; Han et al., 2022), and gene mutations (Stowers and Logan, 2008; Zhang et al., 2020b).

Similar to the findings of previous studies (Zhang et al., 2013; McKenzie et al., 2014; Gu et al., 2015; Zhang L.-W. et al., 2016), we also found that there were 12 RpedOBP and six RpedCSP genes highly expressed in non-antennal tissues, including four leg-biased genes (RpedOBP14, RpedOBP35, RpedOBP44, and RpedCSP6), six head-biased genes (RpedOBP16, RpedOBP29, RpedOBP30, RpedOBP31, RpedOBP45, and RpedOBP46), one thorax-biased gene (RpedOBP39), two abdomen-biased genes (RpedOBP26 and RpedOBP28), and five wing-biased genes (RpedCSP1, RpedCSP2, RpedCSP8, RpedCSP10, and RpedCSP25), indicating that these genes may have other non-olfactory functions.

## Conclusion

In conclusion, we identified 49 OBPs and 25 CSPs in the *R. pedestris* genome and found that these genes were clustered in highly conserved groups comprising OBP and CSP genes from other hemipteran species. To further understand the functions of these genes, we conducted comprehensive and comparative phylogenetic analyses and studied the gene expression profiles of OBPs and CSPs. We found that most RpedOBPs displayed antennal-biased expression, but many RpedCSPs were detected in the antennae and were highly expressed in non-antennal tissues, and some genes showed characteristics of sex-biased expression. Tissue- and sex-biased expression patterns will help us identify the functions of RpedOBPs and RpedCSPs, which will also aid in understanding the olfactory mechanism of *R. pedestris* and the development of environmentally friendly insecticides against this pest in the future.

## Data Availability

The original contributions presented in the study are included in the article/Supplementary Material, further inquiries can be directed to the corresponding authors.

## References

[B1] Bohbot J. Sobrio F. Lucas P. Nagnan-Le Meillour P. (1998). Functional Characterization of a New Class of Odorant-Binding Proteins in the MothMamestra Brassicae. Biochem. Biophysical Res. Commun. 253 (2), 489–494. 10.1006/bbrc.1998.9806 9878563

[B2] Bohbot J. Vogt R. G. (2005). Antennal Expressed Genes of the Yellow Fever Mosquito (Aedes aegypti L.); Characterization of Odorant-Binding Protein 10 and Takeout. Insect Biochem. Mol. Biol. 35 (9), 961–979. 10.1016/j.ibmb.2005.03.010 15978998

[B3] Cao D. Liu Y. Walker W. B. Li J. Wang G. (2014). Molecular Characterization of the Aphis Gossypii Olfactory Receptor Gene Families. PLoS One 9 (6), e101187. 10.1371/journal.pone.0101187 24971460 PMC4074156

[B4] Chen C. Chen H. Zhang Y. Thomas H. R. Frank M. H. He Y. (2020). TBtools: an Integrative Toolkit Developed for Interactive Analyses of Big Biological Data. Mol. Plant 13 (8), 1194–1202. 10.1016/j.molp.2020.06.009 32585190

[B5] Chen G.-L. Pan Y.-F. Ma Y.-F. Wang J. He M. He P. (2018). Binding Affinity Characterization of an Antennae-Enriched Chemosensory Protein from the White-Backed Planthopper, Sogatella Furcifera (Horváth), with Host Plant Volatiles. Pesticide Biochem. Physiology 152, 1–7. 10.1016/j.pestbp.2018.09.006 30497699

[B6] Chen J. H. Cui J. Tang J. W. Bi R. Zhang J. P. Shi S. S. (2018). Effects of Temperature on the Growth and Development of Riptortus Pedestris Fabricius. Chi J. Oil Crop Sci. 40 (4), 579–584. 10.7505/j.issn.1007-9084.2018.04.016

[B7] Cheng T. Wu J. Wu Y. Chilukuri R. V. Huang L. Yamamoto K. (2017). Genomic Adaptation to Polyphagy and Insecticides in a Major East Asian Noctuid Pest. Nat. Ecol. Evol. 1 (11), 1747–1756. 10.1038/s41559-017-0314-4 28963452

[B8] Cui G. Z. Zhu J. J. (2016). Pheromone-Based Pest Management in China: Past, Present, and Future Prospects. J. Chem. Ecol. 42 (7), 557–570. 10.1007/s10886-016-0731-x 27481347

[B9] Cui H.-H. Gu S.-H. Zhu X.-Q. Wei Y. Liu H.-W. Khalid H. D. (2017). Odorant-binding and Chemosensory Proteins Identified in the Antennal Transcriptome of Adelphocoris Suturalis Jakovlev. Comp. Biochem. Physiology Part D Genomics Proteomics 24, 139–145. 10.1016/j.cbd.2016.03.001 27085212

[B10] Dani F. R. Michelucci E. Francese S. Mastrobuoni G. Cappellozza S. La Marca G. (2011). Odorant-binding Proteins and Chemosensory Proteins in Pheromone Detection and Release in the Silkmoth Bombyx mori. Chem. Senses 36 (4), 335–344. 10.1093/chemse/bjq137 21220518

[B11] Elfekih S. Chen C.-Y. Hsu J.-C. Belcaid M. Haymer D. (2016). Identification and Preliminary Characterization of Chemosensory Perception-Associated Proteins in the Melon Fly Bactrocera Cucurbitae Using RNA-Seq. Sci. Rep. 6, 19112. 10.1038/srep19112 26752702 PMC4707516

[B12] Glaser N. Gallot A. Legeai F. Harry M. Kaiser L. Le Ru B. (2015). Differential Expression of the Chemosensory Transcriptome in Two Populations of the Stemborer Sesamia Nonagrioides. Insect Biochem. Mol. Biol. 65, 28–34. 10.1016/j.ibmb.2015.07.008 26316282

[B13] Gong D.-P. Zhang H.-J. Zhao P. Xia Q.-Y. Xiang Z.-H. (2009). The Odorant Binding Protein Gene Family from the Genome of Silkworm, Bombyx mori. BMC Genomics 10, 332. 10.1186/1471-2164-10-332 19624863 PMC2722677

[B14] Gu S.-H. Wang S.-P. Zhang X.-Y. Wu K.-M. Guo Y.-Y. Zhou J.-J. (2011). Identification and Tissue Distribution of Odorant Binding Protein Genes in the Lucerne Plant Bug Adelphocoris Lineolatus (Goeze). Insect Biochem. Mol. Biol. 41 (4), 254–263. 10.1016/j.ibmb.2011.01.002 21232599

[B15] Gu S.-H. Wang S.-Y. Zhang X.-Y. Ji P. Liu J.-T. Wang G.-R. (2012). Functional Characterizations of Chemosensory Proteins of the Alfalfa Plant Bug Adelphocoris Lineolatus Indicate Their Involvement in Host Recognition. PLoS One 7 (8), e42871. 10.1371/journal.pone.0042871 22900060 PMC3416781

[B16] Gu S.-H. Wu K.-M. Guo Y.-Y. Field L. M. Pickett J. A. Zhang Y.-J. (2013). Identification and Expression Profiling of Odorant Binding Proteins and Chemosensory Proteins between Two Wingless Morphs and a Winged Morph of the Cotton Aphid Aphis Gossypii Glover. PLoS One 8 (9), e73524. 10.1371/journal.pone.0073524 24073197 PMC3779235

[B17] Gu S.-H. Zhou J.-J. Gao S. Wang D.-H. Li X.-C. Guo Y.-Y. (2015). Identification and Comparative Expression Analysis of Odorant Binding Protein Genes in the Tobacco Cutworm Spodoptera Litura. Sci. Rep. 5, 13800. 10.1038/srep13800 26346731 PMC4561897

[B18] Han W.-K. Yang Y.-L. Si Y.-X. Wei Z.-Q. Liu S.-R. Liu X.-L. (2022). Involvement of GOBP2 in the Perception of a Sex Pheromone Component in Both Larval and Adult Spodoptera Litura Revealed Using CRISPR/Cas9 Mutagenesis. Insect Biochem. Mol. Biol. 141, 103719. 10.1016/j.ibmb.2022.103719 34999200

[B19] He M. He P. (2014). Molecular Characterization, Expression Profiling, and Binding Properties of Odorant Binding Protein Genes in the Whitebacked Planthopper, Sogatella Furcifera. Comp. Biochem. Physiology Part B Biochem. Mol. Biol. 174, 1–8. 10.1016/j.cbpb.2014.04.008 24815350

[B20] He M. Ma Y.-F. Guo H. Liu X.-Z. Long G.-J. Wang Q. (2022). Genome-wide Identification and Expression Pattern Analysis of Novel Chemosensory Genes in the German Cockroach Blattella germanica. Genomics 114 (2), 110310. 10.1016/j.ygeno.2022.110310 35151840

[B21] He M. Zhang Y.-N. He P. (2015). Molecular Characterization and Differential Expression of an Olfactory Receptor Gene Family in the White-Backed Planthopper Sogatella Furcifera Based on Transcriptome Analysis. PLoS One 10 (11), e0140605. 10.1371/journal.pone.0140605 26540266 PMC4634861

[B22] He P. Chen G. L. Li S. Wang J. Ma Y. F. Pan Y. F. (2019). Evolution and Functional Analysis of Odorant‐binding Proteins in Three Rice Planthoppers: Nilaparvata Lugens Sogatella Furcifera and Laodelphax Striatellus. Pest. Manag. Sci. 75 (6), 1606–1620. 10.1002/ps.5277 30515974

[B23] He X. Tzotzos G. Woodcock C. Pickett J. A. Hooper T. Field L. M. (2010). Binding of the General Odorant Binding Protein of Bombyx mori BmorGOBP2 to the Moth Sex Pheromone Components. J. Chem. Ecol. 36 (12), 1293–1305. 10.1007/s10886-010-9870-7 20981477

[B24] Huang H. J. Ye Y. X. Ye Z. X. Yan X. T. Wang X. Wei Z. Y. (2021). Chromosome‐level Genome Assembly of the Bean Bug Riptortus Pedestris. Mol. Ecol. Resour. 21 (7), 2423–2436. 10.1111/1755-0998.13434 34038033

[B25] Ingham V. A. Anthousi A. Douris V. Harding N. J. Lycett G. Morris M. (2020). A Sensory Appendage Protein Protects Malaria Vectors from Pyrethroids. Nature 577 (7790), 376–380. 10.1038/s41586-019-1864-1 31875852 PMC6974402

[B26] Krieger J. von Nickisch-Rosenegk E. Mameli M. Pelosi P. Breer H. (1996). Binding Proteins from the Antennae of Bombyx mori. Insect Biochem. Mol. Biol. 26 (3), 297–307. 10.1016/0965-1748(95)00096-8 8900598

[B27] Kumar S. Stecher G. Tamura K. (2016). MEGA7: Molecular Evolutionary Genetics Analysis Version 7.0 for Bigger Datasets. Mol. Biol. Evol. 33 (7), 1870–1874. 10.1093/molbev/msw054 27004904 PMC8210823

[B28] Larter N. K. Sun J. S. Carlson J. R. (2016). Organization and Function of Drosophila Odorant Binding Proteins. Elife 5, e20242. 10.7554/eLife.20242 27845621 PMC5127637

[B29] Latorre-Estivalis J. M. Grosse-Wilde E. da Rocha Fernandes G. Hansson B. S. Lorenzo M. G. (2022). Changes in Antennal Gene Expression Underlying Sensory System Maturation in Rhodnius prolixus. Insect Biochem. Mol. Biol. 140, 103704. 10.1016/j.ibmb.2021.103704 34942331

[B30] Leal W. S. (2013). Odorant Reception in Insects: Roles of Receptors, Binding Proteins, and Degrading Enzymes. Annu. Rev. Entomol. 58 (1), 373–391. 10.1146/annurev-ento-120811-153635 23020622

[B31] Li K. Zhang X. Guo J. Penn H. Wu T. Li L. (2019). Feeding of Riptortus Pedestris on Soybean Plants, the Primary Cause of Soybean Staygreen Syndrome in the Huang-Huai-Hai River Basin. Crop J. 7, 360–367. 10.1016/j.cj.2018.07.008

[B32] Li L.-L. Xu J.-W. Yao W.-C. Yang H.-H. Dewer Y. Zhang F. (2021). Chemosensory Genes in the Head of Spodoptera Litura Larvae. Bull. Entomol. Res. 111 (4), 454–463. 10.1017/S0007485321000109 33632348

[B33] Li L. L. Huang J. R. Xu J. W. Yao W. C. Yang H. H. Shao L. (2022). Ligand‐binding Properties of Odorant‐binding Protein 6 in Athetis Lepigone to Sex Pheromones and Maize Volatiles. Pest Manag. Sci. 78 (1), 52–62. 10.1002/ps.6606 34418275

[B34] Li X.-M. Zhu X.-Y. Wang Z.-Q. Wang Y. He P. Chen G. (2015). Candidate Chemosensory Genes Identified in Colaphellus Bowringi by Antennal Transcriptome Analysis. BMC Genomics 16 (1), 1028. 10.1186/s12864-015-2236-3 26626891 PMC4667470

[B35] Li Z.-Q. Zhang S. Luo J.-Y. Cui J.-J. Ma Y. Dong S.-L. (2013). Two Minus-C Odorant Binding Proteins from Helicoverpa Armigera Display Higher Ligand Binding Affinity at Acidic pH Than Neutral pH. J. Insect Physiology 59 (3), 263–272. 10.1016/j.jinsphys.2012.12.004 23295622

[B36] Liu N.-Y. Yang F. Yang K. He P. Niu X.-H. Xu W. (2015). Two Subclasses of Odorant-Binding Proteins inSpodoptera Exiguadisplay Structural Conservation and Functional Divergence. Insect Mol. Biol. 24 (2), 167–182. 10.1111/imb.12143 25345813

[B37] Maleszka R. Stange G. (1997). Molecular Cloning, by a Novel Approach, of a cDNA Encoding a Putative Olfactory Protein in the Labial Palps of the Moth Cactoblastis cactorum. Gene 202 (1-2), 39–43. 10.1016/s0378-1119(97)00448-4 9427543

[B38] Martín-Blázquez R. Chen B. Kang L. Bakkali M. (2017). Evolution, Expression and Association of the Chemosensory Protein Genes with the Outbreak Phase of the Two Main Pest Locusts. Sci. Rep. 7 (1), 6653. 10.1038/s41598-017-07068-0 28751682 PMC5532218

[B39] Matsuo T. Sugaya S. Yasukawa J. Aigaki T. Fuyama Y. (2007). Odorant-binding proteins OBP57d and OBP57e affect taste perception and host-plant preference in Drosophila sechellia. PLoS Biol. 5 (5), e118. 10.1371/journal.pbio.0050118 17456006 PMC1854911

[B40] McKenna M. P. Hekmat-Scafe D. S. Gaines P. Carlson J. R. (1994). Putative Drosophila Pheromone-Binding Proteins Expressed in a Subregion of the Olfactory System. J. Biol. Chem. 269 (23), 16340–16347. 10.1016/s0021-9258(17)34013-9 8206941

[B41] McKenzie S. K. Oxley P. R. Kronauer D. J. (2014). Comparative Genomics and Transcriptomics in Ants Provide New Insights into the Evolution and Function of Odorant Binding and Chemosensory Proteins. BMC Genomics 15, 718. 10.1186/1471-2164-15-718 25159315 PMC4161878

[B42] Missbach C. Vogel H. Hansson B. S. Groβe-Wilde E. (2015). Identification of Odorant Binding Proteins and Chemosensory Proteins in Antennal Transcriptomes of the Jumping BristletailLepismachilis Y-Signataand the FirebratThermobia domestica:Evidence for an Independent OBP-OR Origin. Chemse 40 (9), 615–626. 10.1093/chemse/bjv050 26377345

[B43] Muller P. Y. Janovjak H. Miserez A. R. Dobbie Z. (2002). Processing of Gene Expression Data Generated by Quantitative Real-Time RT-PCR. Biotechniques 32 (6), 1372–1379. 12074169

[B44] Paula D. P. Togawa R. C. Costa M. M. C. Grynberg P. Martins N. F. Andow D. A. (2016). Identification and Expression Profile of Odorant‐binding Proteins in Halyomorpha Halys (Hemiptera: Pentatomidae). Insect Mol. Biol. 25 (5), 580–594. 10.1111/imb.12243 27170546

[B45] Pelosi P. Calvello M. Ban L. (2005). Diversity of Odorant-Binding Proteins and Chemosensory Proteins in Insects. Chem. Senses 30 (Suppl. 1), i291–i292. 10.1093/chemse/bjh229 15738163

[B46] Pelosi P. Iovinella I. Zhu J. Wang G. Dani F. R. (2018). Beyond Chemoreception: Diverse Tasks of Soluble Olfactory Proteins in Insects. Biol. Rev. 93 (1), 184–200. 10.1111/brv.12339 28480618

[B47] Petersen T. N. Brunak S. von Heijne G. Nielsen H. (2011). SignalP 4.0: Discriminating Signal Peptides from Transmembrane Regions. Nat. Methods 8 (10), 785–786. 10.1038/nmeth.1701 21959131

[B48] Poivet E. Gallot A. Montagné N. Glaser N. Legeai F. Jacquin-Joly E. (2013). A Comparison of the Olfactory Gene Repertoires of Adults and Larvae in the Noctuid Moth Spodoptera Littoralis. PLoS One 8 (4), e60263. 10.1371/journal.pone.0060263 23565215 PMC3614943

[B49] Qin Y. G. Yang Z. K. Song D. L. Wang Q. Gu S. H. Li W. H. (2020). Bioactivities of Synthetic Salicylate‐substituted Carboxyl ( E )‐β‐Farnesene Derivatives as Ecofriendly Agrochemicals and Their Binding Mechanism with Potential Targets in Aphid Olfactory System. Pest Manag. Sci. 76 (7), 2465–2472. 10.1002/ps.5787 32061021

[B50] Renou M. Anton S. (2020). Insect Olfactory Communication in a Complex and Changing World. Curr. Opin. Insect Sci. 42, 1–7. 10.1016/j.cois.2020.04.004 32485594

[B51] Rihani K. Ferveur J.-F. Briand L. (2021). The 40-Year Mystery of Insect Odorant-Binding Proteins. Biomolecules 11 (4), 509. 10.3390/biom11040509 33808208 PMC8067015

[B52] Robertson H. M. (2019). Molecular Evolution of the Major Arthropod Chemoreceptor Gene Families. Annu. Rev. Entomol. 64, 227–242. 10.1146/annurev-ento-020117-043322 30312552

[B53] Schultze A. Schymura D. Forstner M. Krieger J. (2012). Expression Pattern of a 'Plus-C' Class Odorant Binding Protein in the Antenna of the Malaria Vector Anopheles gambiae. Insect Mol. Biol. 21 (2), 187–195. 10.1111/j.1365-2583.2011.01125.x 22211989

[B54] Sengul M. S. Tu Z. (2010). Expression Analysis and Knockdown of Two Antennal Odorant-Binding Protein Genes inAedes Aegypti. J. Insect Sci. 10, 1–18. 10.1673/031.010.14131 21062207 PMC3016889

[B55] Simon P. (2003). Q-Gene: Processing Quantitative Real-Time RT-PCR Data. Bioinformatics 19 (11), 1439–1440. 10.1093/bioinformatics/btg157 12874059

[B56] Spinelli S. Lagarde A. Iovinella I. Legrand P. Tegoni M. Pelosi P. (2012). Crystal Structure of Apis mellifera OBP14, a C-Minus Odorant-Binding Protein, and its Complexes with Odorant Molecules. Insect Biochem. Mol. Biol. 42 (1), 41–50. 10.1016/j.ibmb.2011.10.005 22075131

[B57] Stowers L. Logan D. W. (2008). LUSH Shapes up for a Starring Role in Olfaction. Cell 133 (7), 1137–1139. 10.1016/j.cell.2008.06.010 18585346

[B58] Sun Y. Qiao H. Ling Y. Yang S. Rui C. Pelosi P. (2011). New Analogues of (E)-β-Farnesene with Insecticidal Activity and Binding Affinity to Aphid Odorant-Binding Proteins. J. Agric. Food Chem. 59 (6), 2456–2461. 10.1021/jf104712c 21341697

[B59] Vogt R. G. (2005). “Molecular Basis of Pheromone Detection in Insects,” in Comprehensive Insect Physiology, Biochemistry, Pharmacology and Molecular Biology Endocrinology. Editors LI Gilbert K. Iatro Gill S. S. (London: Elsevier), 753–803. 10.1016/b0-44-451924-6/00047-8

[B60] Vogt R. G. Riddiford L. M. (1981). Pheromone Binding and Inactivation by Moth Antennae. Nature 293 (5828), 161–163. 10.1038/293161a0 18074618

[B61] Wan F. Yin C. Tang R. Chen M. Wu Q. Huang C. (2019). A Chromosome-Level Genome Assembly of Cydia Pomonella Provides Insights into Chemical Ecology and Insecticide Resistance. Nat. Commun. 10 (1), 4237. 10.1038/s41467-019-12175-9 31530873 PMC6748993

[B62] Xu H. Zhao J. Li F. Yan Q. Meng L. Li B. (2021). Chemical Polymorphism Regulates the Attractiveness to Nymphs in the Bean Bug Riptortus Pedestris. J. Pest Sci. 94, 463–472. 10.1007/s10340-020-01268-w

[B63] Xue J. Zhou X. Zhang C.-X. Yu L.-L. Fan H.-W. Wang Z. (2014). Genomes of the Rice Pest Brown Planthopper and its Endosymbionts Reveal Complex Complementary Contributions for Host Adaptation. Genome Biol. 15 (12), 521. 10.1186/s13059-014-0521-0 25609551 PMC4269174

[B64] Yang K. He P. Dong S.-L. (2014). Different Expression Profiles Suggest Functional Differentiation Among Chemosensory Proteins in Nilaparvata Lugens (Hemiptera: Delphacidae). J. Insect Sci. 14 (1), 270. 10.1093/jisesa/ieu132 25527582 PMC5657923

[B65] Yi X. Qi J. Zhou X. Hu M. Y. Zhong G. H. (2017). Differential Expression of Chemosensory-Protein Genes in Midguts in Response to Diet of Spodoptera Litura. Sci. Rep. 7 (1), 296. 10.1038/s41598-017-00403-5 28331183 PMC5428418

[B66] Zhang L.-W. Kang K. Jiang S.-C. Zhang Y.-N. Wang T.-T. Zhang J. (2016). Analysis of the Antennal Transcriptome and Insights into Olfactory Genes in Hyphantria cunea (Drury). PLoS One 11 (10), e0164729. 10.1371/journal.pone.0164729 27741298 PMC5065180

[B67] Zhang L. Guo M. Zhuo F. Xu H. Zheng N. Zhang L. (2019). An Odorant-Binding Protein Mediates Sexually Dimorphic Behaviors via Binding Male-specific 2-heptanone in Migratory Locust. J. Insect Physiology 118, 103933. 10.1016/j.jinsphys.2019.103933 31449797

[B68] Zhang Y.-N. Jin J.-Y. Jin R. Xia Y.-H. Zhou J.-J. Deng J.-Y. (2013). Differential Expression Patterns in Chemosensory and Non-chemosensory Tissues of Putative Chemosensory Genes Identified by Transcriptome Analysis of Insect Pest the Purple Stem Borer Sesamia Inferens (Walker). PLoS One 8 (7), e69715. 10.1371/journal.pone.0069715 23894529 PMC3722147

[B69] Zhang Y.-N. Kang K. Xu L. Zhu X.-Y. Qian J.-L. Zhang Z.-J. (2017a). Deep Sequencing of Antennal Transcriptome from Callosobruchus Chinensis to Characterize Odorant Binding Protein and Chemosensory Protein Genes. J. Stored Prod. Res. 74 (74), 13–21. 10.1016/j.jspr.2017.08.006

[B70] Zhang Y.-N. Xu J.-W. Zhang X.-C. Zhang X.-Q. Li L.-L. Yuan X. (2020a). Organophosphorus Insecticide Interacts with the Pheromone-Binding Proteins of Athetis Lepigone: Implication for Olfactory Dysfunction. J. Hazard. Mater. 397, 122777. 10.1016/j.jhazmat.2020.122777 32388456

[B71] Zhang Y.-N. Ye Z.-F. Yang K. Dong S.-L. (2014). Antenna-predominant and Male-Biased CSP19 of Sesamia Inferens Is Able to Bind the Female Sex Pheromones and Host Plant Volatiles. Gene 536 (2), 279–286. 10.1016/j.gene.2013.12.011 24361960

[B72] Zhang Y.-N. Zhang X.-Q. Zhang X.-C. Xu J.-W. Li L.-L. Zhu X.-Y. (2020b). Key Amino Acid Residues Influencing Binding Affinities of Pheromone-Binding Protein from Athetis Lepigone to Two Sex Pheromones. J. Agric. Food Chem. 68 (22), 6092–6103. 10.1021/acs.jafc.0c01572 32392414

[B73] Zhang Y.-N. Zhu X.-Y. Ma J.-F. Dong Z.-P. Xu J.-W. Kang K. (2017b). Molecular Identification and Expression Patterns of Odorant Binding Protein and Chemosensory Protein Genes inAthetis lepigone(Lepidoptera: Noctuidae). PeerJ 5, e3157. 10.7717/peerj.3157 28382236 PMC5376112

[B74] Zhang Y.-N. Zhu X.-Y. Zhang Q. Yin C.-Y. Dong Z.-P. Zuo L.-H. (2016). De Novo assembly and Characterization of Antennal Transcriptome Reveal Chemosensory System in Nysius Ericae. J. Asia-Pacific Entomology 19, 1077–1087. 10.1016/j.aspen.2016.09.013

[B75] Zhou J.-J. (2010). Odorant-binding Proteins in Insects. Vitam. Horm. 83, 241–272. 10.1016/S0083-6729(10)83010-9 20831949

[B76] Zhou S.-S. Sun Z. Ma W. Chen W. Wang M.-Q. (2014). De Novo analysis of the Nilaparvata Lugens (Stål) Antenna Transcriptome and Expression Patterns of Olfactory Genes. Comp. Biochem. Physiology Part D Genomics Proteomics 9, 31–39. 10.1016/j.cbd.2013.12.002 24440828

[B77] Zhou W. Yuan X. Qian P. Cheng J. Zhang C. Gurr G. (2015). Identification and Expression Profiling of Putative Chemosensory Protein Genes in Two Rice Planthoppers, Laodelphax Striatellus (Fallén) and Sogatella Furcifera (Horváth). J. Asia-Pacific Entomology 18 (4), 771–778. 10.1016/j.aspen.2015.09.006

[B78] Zhou X.-H. Ban L.-P. Iovinella I. Zhao L.-J. Gao Q. Felicioli A. (2013). Diversity, Abundance, and Sex-specific Expression of Chemosensory Proteins in the Reproductive Organs of the Locust Locusta migratoria Manilensis. Biol. Chem. 394 (1), 43–54. 10.1515/hsz-2012-0114 23096575

[B79] Zhu G.-H. Xu J. Cui Z. Dong X.-T. Ye Z.-F. Niu D.-J. (2016). Functional Characterization of SlitPBP3 in Spodoptera Litura by CRISPR/Cas9 Mediated Genome Editing. Insect Biochem. Mol. Biol. 75, 1–9. 10.1016/j.ibmb.2016.05.006 27192033

[B80] Zhu J. Iovinella I. Dani F. R. Liu Y.-L. Huang L.-Q. Liu Y. (2016). Conserved Chemosensory Proteins in the Proboscis and Eyes of Lepidoptera. Int. J. Biol. Sci. 12 (11), 1394–1404. 10.7150/ijbs.16517 27877091 PMC5118785

