# Supplementary material for: Genome-Wide Analysis of Odorant-Binding Proteins and Chemosensory Proteins in the Bean bug Riptortus pedestris
Source: Front Physiol. 2022 Jul 14;13:949607. doi: 10.3389/fphys.2022.949607 (PMC9329939; doi:10.3389/fphys.2022.949607)
Supplement: Supplementary file 3 [file Table2.DOC]

**Table S2. The sequences of OBPs and CSPs for phylogenetic tree**

>LlinOBP1

GELPEEMREMAQGLHDGCVEETGVDNGLIGPCAKGNFADDQKLKCYFKCVFGNLGVISDEGELDAEAFGSILPDNMQELLPTIRGCAGTTGADPCELAMNFNKCLQKVDPVNFMVI

>LlinOBP2

YMSQAQMKQAMKTVRNMCIPKSGVAKEALAKMVEGEFDDSDQKLKCYLGCVLGMMQAVKNNKINLTMVRNQISKMLAPEQGQRILTAFEGCATVTGDDNCDLAFKFAKCIYDTDKELLFQAFIVP

>LlinOBP3

YQEVLKATLQDCKGGKEITQEEVDEFVKPLIPKNEEERCLMACVFRAYNVIVDGHFDPKLAYGVAKNILHENPEKLKHIKETLDYCGHEIPTKMDNECDLAGEVMACRNKYNKDHGYDQDP

>LlinOBP4

YQEQLKQTIKDCQGGKEVTDDELEEFTKPLIPKNEEERCIMACVMRTYNIINNGHYDPKIAFGILKGILKDHPEKLNKIKEVMDHCGEDVPQHMDNECDLAGEIMQCEVKYQKAMGLA

>LlinOBP5

YQEQLKATIQKCQDGREVTDDEVEEFTKPLIPKNEEERCLVACVFKEYKVIIDGHFDPVNALNVAKMVYKEYPEKWERIRDVIDHCGEDIPTHNDNECDLAGDIMKCEVKYLNSMPKITSLELLAGSIAATEEP

>LlinOBP6

NEKKANEKVTEIFNKCKETWPVTDEEIEQVKQKQSIPESKNVKCILACMLKEAKILRDGEYNKENAELMADVLYKDEPEHAXKSKQIIEMCSAELGTKTEGDDCEYAYKMSVCASKHAKELGVKTPEF

>LlinOBP7

VKANEKKANEKVTEIFNKCKETWPVTRRGNXTSENRSRAFPNPKNVKCILACMLKEAKILRDGEYNKENAELMADVLYKDEPEHAEKSKQIIEMCSAELGTKTEGDDCEFAFQGGQLKIMENSFTLRSLLADCLNRRYPGHVGYRYL

>LlinOBP8

DEQTNAMVAKAFNKCHGEFPIGDDEMKGVREKSTVPDSHNAKCLMACMLKEGKILRDGKYEKENAIVMADVLNKDDPAAADKAKQLVETCATQVGSDASADECEFAYKMAVCAAGEAKKLGVRPPDF

>LlinOBP9

DEQTNAMVAKAFNKCHGEFPIGDDEMKGVREKSTVPDSHNAKCLMACMLKEGKILRDGKYEKENAIVMADVLNKDDPAAADKAKQLVETCATQVGSDASADECEFAYKMAVCAAAWSSSTRFLKTHPFLLQLCTHRSTWHTFKYEYLPGNTSSRDLTHHINIVCVT

>LlinOBP10

NTKELSPVEVYKHKIHEECIKETKATPEQAKIVFNYKDVPKDDGEKCFMECVYKKSGGIDANGKYSIEGFNKLVDMKYKGEENAGAKMIVKDCSSKVAPKEGEKCSVGRTIRECLSAASKENEFFTI

>LlinOBP11

APSANVKEIVQNVSKKCVAETKASPEQAKIAVSQHIPKDDVERCYLQCVYTGVGVIKDGKFSEEGGKKLVALRFHDAKEKELANKLIATCAKEIKAKDGEKCSLGRAVRECFVNHGKQVNFFPSA

>LlinOBP12

MVAECPAYSWVCQSTLSYYLHLQQTLQYIDRDPTLAMNQSSCILTLALTIFVMVVVSGFKELDSVLPQAKQEECRKESNFQGELSGDVSQNVTQELKCFAACSLVKLGLMNEKDGTINTTQLDELIAKHTAGKDAADMFKHSVVEPCLKEVNKTADYCEYSFQLVTCGMNKVKPPTTG

>LlinOBP13

ITPELDKKAKEAVAKCADVPGINEAKKEDCYAACFMTEMGYMTDGKINVENMEEANKQKWDDQQMINKGIEIDKTCAKQVGDTKGKSECAIGYDFGVCKTRLVKANCILRSILQTQLVPSPWWAHLPPLTPLKDSSTFNASFLKGVYREDMEQRNYNKTGLQPPTPLKQ

>LlinOBP14

MKIAFVVSVLVVLATVSAITPELDKKAKEAVAKCADVPEINEAKKEDCYAACFMTEMGYMTDGKINVENMEEANKQKWDDQQMINKGIEIDKTCAKQVGDTKGKSECAIGYDFGVCKTRLVKANCILRSILQXQLVPSPWWAHLPPLTPLKDSSTFNASFLKGVYRGDMEQRNYNKGKNKTGLQPPTPLKQ

>LlinOBP15

ITPELDKKAKEAVAKCADVPGINEAKKEDCYAACFMTEMGYMTDGKINVENMEEANRQKWDDQQMINKGIEIDKTCAKQVGDTKGKSECAIGYDFGVCKTRLVKATGLQPPTPLKQ

>LlinOBP16

ITDELRKKATEARLKCKQQVGLSDKEYQDWVKGISLPITNGGSCCEVCACWMRELGYMTDGHLNLNNMKNVNTQKWSEKANVEKANQIDTLCTARVVQDGRKECEIALDYRKCKTEMIKQNGGPPKPGST

>LlinOBP17

ITDELRKKATETRLKCKQQVGLSDKEYQDWVKGISLPTTNGGSCCEVCACWMRELGYMTDGHLNLNNMKKRQDKYKHS

>LlinOBP18

MRSTGSECFEEVDAKLGNKTSWESDMDPYNCEKVKRMKKRHYCLHECKAKKLGVANEEGVLDFPKVKDLLLSRVNETWQKDILGQAADTCANSKFDQTWKDDTEEYKCNPQAIQFKHCVWKQVEMKCPEEHQNTGRHCKKLRSKISSETSKDSTAKETSV

>LlinOBP19

VTKEYHDRAVAAKDKCAKEHNIKESEIQEFVKKHKLPETEDGKCMIACYMEEMKLITDGKVNVDEWKKSNKEKWDEEAHVAMADEIVDKCNEQVSPDGLAKCEYGFKLTECGLKHRLEKGLPAPNMDDVKRR

>LlinOBP20

MKLVKDDKARPPKPEGYECIDDCIMAKNGFLGTDKKIDAAKVNAAAKTSYTGEWAEPGAKMVEKCLAQVSANKEKGECTSGADIFSICMFRESFINCPEKSWTSSETCKANKERLIKCPKSIPFLNKSAK

>LlinOBP21

QMDEDPDCRPPHPPGKEAQCCPLPDFVGVVDNFHDVMHKCSDEAGLRKPSGPPGSGTPPTAEEMAAHMSAHECADECLFKNTKYLQSNGELDKDAIKASVTKIFTGDWAALASSAADKCLASAKSEVGASAKCKSGARQMVKCFTRAMFLNCPASSWTESTECAAAKARITKCPNAMVPMIPPYPQPISANSTILDSLFACITDGEYMEFQSISSGRLG

>LlinOBP22

MPSQLPCPFFLLNIAFAHPGHFDEDPECRQPHHHRHEENDCCKVPSLFSNNKDEMHELVHKCFEEAGIKKHGPHHEHHGPPPLEDGPIPPPPPPPFSPKNDSKLDCVEQCFLKNLDLVDDEGDLKVDDLKALVTEKFSGDWASVGSSAIEKCLEKAKTEENESSKCKAGSKRVLHCHCTQHASYPKMGGSQSARKVTLDNPNPDPHDLNNVI

>LlinOBP23

HPGHFDEDPECRQPHHHRHEENDCCKVPSLFSNNKDEMHELVHKCFEEAGIKKHGPHHEHHGPPPLEDGPIPPPPPPPFSPKNDSKFDCVEQCFLKNLDLVDDEGDLKVDDLKALVTEKFSGDWASVGSSAIEKCLEKAKTEENEPSKCKAGSKRVLHCLAREFFMNCPASDWTESEVCLAAKDRVSKCPHSLPPMHH

>LlinOBP24

MADSVGECMKLIKVKPEKGPPVPEGFDCMDTCVFSKLGFIGADNKLDPEKLAKKFSELFKGDWSALSESTLKKCLPMADVGKGVCSSGADVFKFCLIRELYMNCPASSWTKSDLCKANVERLEKCPNSLPFMNGSGLKNKSSR

>LlinOBP25

QMDEDPECRPPHPPGKAGDCCVQPKLFDEGDMPDVIKKCHEEAGVKRPSGPPGSGTPPTAEEMAAHKSAHECAAECIFKNNNFIKSDGELDXDAIKATVTKMFTGDWATLASTTIDKCLASAKSEVDASPKCKSGADQVVRCFGRSLFIGCPASAWTESTECAAEKARLTKCPNAMPPPPHHKH

>LlinOBP26

FKELDSVLPQAKQEECRKESNFQGELSGDLSQNVTQELKCFAACSLVKLGLMNEKDGTINTTQLDELIAKHTEGKDAADMFKHSVVEPCLKEVNKTADYCEYSFQLVTCGMNKVKPPTTG

>LlinOBP27

KFYRRFKHHQIAAHLTFCVYVKTGSRDKTETPRSERPLCKAPTSAPRKLEKVINQCQEEIKYALLQEALSVLGETVSLRTALTRNRSKRETFTGEERRIAGCLLQCVYRKMKALDETGFPTATGLVKIYSEGVEDRNYYLATIQGVQQCLSRELQNRNKNPSIVKAEGYSCDVAYDVFNCVSEEIEQLCGTSP

>LlinOBP28

VDEKRPLCKAPTSAPRKLEKVINQCQEEIKYALLQEALSVLGETVSLRTALTRNRSKRETFTGEERRIAGCLLQCVYRKMKALDETGFPTATGLVKIYSEGVEDRNYYLATIQGVQQCLSRELQNRNKNPSIVKAEGYSCDVAYDVFNCVSEEIEQLCGTSP

>LlinOBP29

QQEDCKTAPAGWPKRPPQCCDLPFPLEGMKREFGSCIRQIGNRQSSAVPTAQAVRDARLCIEECVYKGLGFMEEHNLNKDQLLEQLKKGVAGKKDWEKPMEDAVKSCHETITKRETPQEGACQDSAHEFTHCVMRQLFLSCPASEWNNNDECNLVKNRMQACPNIPPPPPPPPQGFRGQGPPQPQ

>LlinOBP30

NPTTPNPTSSSHAASVSGGSTVSGVSKSPEEVKQKIKEQVEALTGACKSQTKITGEQAKIVATQAIPKTEAEKCFLECIYTGLQLTKDGKFNEPAARALAQKRFGNAPEDLTKANSMIDTCVKEVVVKDLNEKCALGRLIRECFVKNGAKINFFPKP

>LlinOBP31

QMDEDPDCRPPHPPGKEAQCCPLPDFVGVVDNFHDVMHKCSDEAGLRKPSGPPGSGTPPTAEEMAAHMSAHECADECLFKNTKYLQSNGELDKDAIKASVTKIFTGDWAALASSAADKCLASAKSEVGASAKCKSGARQMVKCFTRAMFLNCPASSWTESTECAAAKARITKCPNAMVPMIPPKH

>LlinOBP32

MEVNAERNVTDEQRXAVRLCSRYTEVESGLAEAGYDCLAECFFIKLGLMGEDKTLNKENILEEVRIQFHEDXVEPARKALETCMEKKYNTKCPSGIDGTMQCFTVQLMLNCPXQNWTDGEECKETRTFMEKCGETLNYYD

>LlinOBP33

LEKVINQCQEEIKYALLQEALSVLGETVSLRTALTRNRSKRETFTGEERRIAGFEFRCDLXDGWKWSQEERGGSSPTWFEINTIPGMNTTSRSWRLSSQISSSSVFCNVYTGK

>AlinOBP13

LVSGHRALDGILPQANQDECREESNFRGELNDDVGRNVTQELKCFAACSLMKLGIMNEKDGTVNMTRLDELIASHTPGKDAADVFKTTVVEPCMKEVKKSTDYCEYSYQLIACGMSKVP

>AlinOBP11

ISKEYHDKAIEAKNTCAKLHNVDDETIMTYWKNHQLPEKEPETCIVICYLKEMKLVVDGKVDADAWKASNKEKWDDEKHVAAADEIVDKCSAEVPPTENECEWGLALTKCALKHGKEAGIPPPDMEHPKRR

>AlinOBP9

SQRTKQQPKSKTKESVVGATRPRDAKATECVNKVNANEEESASFFRKEIPETEAGKCLLACYLEGKGLIVGGKISSSGAARVAARAYPNNRVKTGNVKHILSHCGTIAGRESNNCEMAYKLADCTTTLSDKFRL

>AlinOBP6

KELTDEQKEQIFAEIKNCMESTKLTDEEFESIMAKKELPTSIEGKCFTKCLMEKMEYLEEGGKINVIAVQAGMEENMEKESEITKAKEVIQQCADSVPPEDSCEYAYGISQCMYNKMKEAGISGS

>AlinOBP4

GELPEEMKEMAQGLHDSCVEETGVDNGLIAPCAKGNFADDAKLRCYFKCVFGNLGVISDEGELDAEAFGSILPDSMQELLPTIKSCGGTTGSDPCDLAMNFNKCLQKADPVNFLVI

>AlinOBP2

YQEQLKQTIRDCQDGKEVTDDELEEFTKPLIPRNREEKCIMACVMRTYNIISNGHYDPKIAFGILKGILKDHPEKLNKIKEVMDHCGEDVPSHMDDECDLAGEIMQCEVKYQKAMGMA

>AlinOBP12

MTTKLRSIGLVFIVSISYAFAYQELLKETIKKCQNGRDVTDDEVEEFTKPLVPKNEEERCLVACVFKEYKVIIDGHFDPVNALNVAKVVYKDYPDKVERIKDVLDHCGEDIPTHNDNECDLAGDIMKCEVKYLNSVPKMTSLEFLAGSMAATAEP

>AlinOBP10

QELPPPGDVKNKTVVFKNSFLRSAKYCSSIYETSTLAIMALLMSEKSDDQNGKCFLNCMLQRYRLMSQDGSYNKDKFKPFLEYIPDSKFLQSIRGNLKNCISEKDPDPCEKASKFIKCFYTRARNKGEIGASKEVIPADGF

>AlinOBP8

VINKDYLEKVVTAKDKCLKEFNVDDSVVEDFIVKYNKPQSESGKCMVACFMEERGMMKDGKTITEQVMLDNQEKWIAATHVNMGKEVIDTCDKEVPNEENDKCDLAVDYMMCLVKRGDEAGLPKMDVAQLKH

>AlinOBP5

AMSQAQMKQAMKTVRNMCIPKSGVDKEALAKMVNGEFDESDQKLKCYLGCVLGMMQAVKNNKINLTMVRNQITKMLAPERGQRILAAFESCATVTGDDNCGLAFRFAKCIYDTDKEAFIVP

>AlinOBP3

ISKEYSARMIAAKEKCQKEFNVTDSVVEDFMKRNIKPESKSGKCMVHCIMEEMGMIDDHKINTEQVKLGNKEKWDDPALVELANQVADTCDQEVFTEGRCKCLVAVEYMMCLATHGDEVGLPHVDFEDSQDS

>AlinOBP1

DEQTNAMVAKAFNKCREEFPISDDEIGGVREKTTIPESHNAKCLMACMLREGKMLRDGKYEKENALIMADVLNKDDPASADKAKQLVETCAGKVGTDAGGDECEFAYKMAVCAAEEAKKLGVRPPDF

>AlinOBP7

QQEDCKTAPAGWPRRPPQCCDLPFPLEGMKKEFGSCIRQIGNRQSSAVPTAQAVRDARLCIEECVYKGLGFMDEHKLNKDQLLEQLKKGIADKKDWTKPMEGAVKRCHETITKRETPQEAACQDSAHEFTHCAMRELFLNCPASEWNNNDECNLVKSRMQACPNIPPPPPPPPQGFRGQGPPPQ

>AlinOBP14

MKPPGPPGSGTPPTAEERAARKIAHECADECLYKSSNLLTSAGELDKDAIKALVTKLYTGDWATAATTAIDKCLASAKGEVEATSKCKSGSFQLSRCFMRSMFLGCPASSWTESTECAAAKARLTKCPNAMAPMPHKK

>AfasOBP11

ISKEYHDKAVAARTTCAKLHNVDDKTIMEHWKNHQLPEKEPETCIIICYLKEMKLVVDGKVDADAWKASNKEKWDDEKHVAAADEIVDKCSAEVPPTENECEWGLALTKCGLKHGKEAGIPPPDMEHPKRR

>AsutOBP12

MFQAFVYQKFLKETIKKCQKGKNVTDDEVEEFTKPLVPKNEKERCLVACLFKEYKVIIDGLFVPFNALNVAKVVYKDYPDKVERIKDVLDHCGEDIPSHNDNECDLAGDIMKCEVKYLNSIPKMTSLEFLAGSMAATAEP

>AsutOBP11

AYHDKAIAAKNTCAKLHNVDDETIMKFWKAHQLPEKEPETCIIICYLKEMKLVVDGKVDADAWKASNKEKWDDEKHVAAADEIVDKCSAEVPPTENECEWGLALTKCALKHGKEAGIPPPDMEHPKRR

>AsutOBP8

EGNINKEYLDKLIAAKEKCVKEFSVDDSIVEDLYVRYNKPPTESGKCMVACYMEERGMMKDGKTITEQVMLDNQEKWIAATHVNMGKEVIDTCDKEVPNEENDKCDLAVDYMMCLVKRGDEAGLPKMDVAQLKH

>AsutOBP6

KELTDEQKEQIFAEIKNCMESTKLTDEEFESIMAKKELPTSKEGKCFTKCLMEKMEYLEEGGKINVIAVQAGLEENMEKESEITKAKEIIQQCADTVPPEDSCEYAYGISQCMYTKMKEAGISGGP

>AsutOBP10

QELPPPGDVKNKTVVFKNSFLRSAKYCSSIYETSTLAIMALLMSEKSDDQNGKCFLNCMLQRYRLMSQDGSYNKDKFKPFLEYIPDSKFLQPIRGNLKNCISEKDPDPCEKASKFVKCFYTRARNKGEIGASKEVIPADG

>AsutOBP7

QQEDCKTAPAGWPRRPPQCCYLPFPLEGMKKEFGSCIRQIGNRQSSAVPTAQAVRDARLCIEECVYKGLGFMDEHKLNKDQLLEQLKKGIADKKDWTKPMEGAVKKCHETITKRETPQEAACQDSAHEFTHCVMRELFLNCPASEWSNNDECNLVKSRMQACPNIPPPPPPPPQGFRGQGPPPQ

>AlucOBP1

EVINEECKDRNQSSTEYETFYNCCDLESSFNETKSKEKEEAREFCENEFEKANNVSEDEGPSPASVGQDCYFECVLKKIGAMSEDYKMDKEKVTKWFMEGSHKDFEEVGKQAMEKCYDKTYSKKYCASGVMGLLWCYSEEIVMNCPAKYWDQSEKCTAAKAYMKKCSTNPWRSED

>AlucOBP2

MRSTGSECFEEIDAKLGNKTSLESDMDPYNCEKVKRMKKRHYCMHECKAKKLGVATEEGNLEFPKVKELLLSRVNETWQKDILGQAADTCATSKFDQTWKDDTEEYKCNPQALQFKHCVWKQVEMKCPEEHQNTGRHCKKLRSKISSETSKDIAKETSV

>AlucOBP3

QPDEDPECRPPHPPGKDDKCCTIPELIVGENMQAMMKQCFEESGMERRPPGPPGPPGSGTPPTPEEIEAHRSAHECVDECFFKAAKFMNSDGEFDLEAMKTAAASVFTGDWAPLGSETIDECFASAKSQVSASAKCTSGAHQAKKCILRNFIINCPPSAWNDSTDCAALKARLTKCPNAMPPFPHHKH

>AlucOBP4

VEEGRPLCKAPTTAPRKLEKVINQCQEEIKYALLQEALSVLGETVSLKTALTRNRSKRETFTGEERRIAGCLLQCVYRKMKALDETGFPTATGLVKIYSEGVEDRNYYLATIQGVQQCLSRELQSRNTNPSIVKAEGYSCDVAYDMFNCVSEQIEQLCGTSP

>AlucOBP5

INSIIVLCLVASAVALSQGNPTTPNPSTSHVSSSAGITVSGVSKSPEEIKLKIKEQVATLTGACKTQTKLTGEQAKIVASQAIPKTEAEKCFLECIYQGLQLTKDGKFNEPAARALAQKRFGNAPEDLQKANTMIDICVKEVVVKDENEKCALGRLIRECFVKNGAKINFFPKP

>AlucOBP6

APPEEPAECKIPESDSAELVKCCKLNVVLDEMADSVGECMKLVKGKPEKGPPVPEGFDCMDTCVFSKLGFVANNKLDAEKLTKKFSELFKGDWSALSDSTLKKCLPMAEGAKGSCASGADVFKFCIVRELYMNCPASSWTKSDLCKANVERLEKCPHSMPFLPGTGIKKN

>AlucOBP7

EEQANALVAKAFNKCFGEFPLGDDEMKEVKDKSTVPSSHNAKCLMACMLKEGRILRGGKYELENAILMADVLNKNDHAAADKAKQLIETCAAQVGTDASADECEFAYKMALCASDEAKKLGVRPPDF

>AlucOBP8

VMTQAQMKQAMKTVRNMCIPKSGVDKEALAKMVEGEFDESDQKLKCYLGCVLGMMQAVKNNKINLTMVKNQISKMLAPEQGQRILAAFEGCATVTGDDNCDLAFKFAKCIYDTDKELLFQAFIVP

>AlucOBP9

ITKEYHDRAVAAKDACLKKHPSIKESDVQEFLKKHKLPETDDGKCMIACYMEEMNLMADGKINVEEAKKTNSDKYDGEPDNKELADKLIDHCSSQVSPDGMSKCEYAYQFSKCGLEYGMKNGLTPPKMYEEQRR

>AlucOBP10

MTYHVFFRKFDLPRISRRVRQCYYHSVPRSLSGSSRRMLEETSQHHPKRRSRVSEKHKLPETDDGECMIACYMEEKNLMADGKINVKEANQTNSDKYDGEPDNKQLAEKLIDHCSSQVSPDGMSKCEYAYQFSKCGLEYGMKNGLTPPKMYEEQRR

>AlucOBP11

APTDDMAACMQITNEDSASMATCCDYVIPFSNKTMTTCDKKETSGEMSKEFECVQDCLFSSDNVLGADKKFDPVAWRKHATNTISGDWKEVIANSGSNCEGFKKVLAQSMEKKCPTSESDVSFNCMTLQWYMNCPKSAWTSSESCEASKKKLMSCFGPIFENTS

>AlucOBP12

EINEECKDIENLKTQLENFYGCCDFESMIERVVRTAEEVETDRFCREERKKINSTDGKMPLASEGHDCFMECVLKRMGAMGQDFKFIREKLDDFFLRGYPEEVKQAGKLAFDKCLSKNFSKKYCASGINGLMMCLPEELVMNCPANIWSSHESCPIAKEAIKKCPSYRVMIEQE

>AlucOBP13

FKELDDVLPKPKQDECRKESNFQAELPSDINQNITQELKCFAACSLVKLGLMNEKDGTINMAQLEDLIAKHTGGKDAADMFKHTVVEPCMKEVNKTTDYCEYSFQLVKCGMSKVKPPSTGTEG

>AlucOBP14

YQEVLKATLKDCKGGKEITQEEVDEFMKPLIPKNEEERCLMACVFRAYNVIVDGHFDPKLAYGVAKNILHENPEKLKHIKETLDYCGHEIPTKMDNECDLAGEVMSCRNKYNIDHGYDQDP

>AlucOBP15

ITPELDKRAKAAVAKCADVPRTDEAKKEDCHAGCFMSAMGYMTNGEINVKNMEEANKQKWDDQEIIKKGIQVDTTCAKQVGDTKGKSECTIGYEFSTCKKELVKKVGLPPPTPLKE

>AlucOBP16

ITDELKQKAQAARLTCKQQVGLSDKEFNDWVKGIALPTTDGGTCCEVCACWMRELGYLTGGRVNLENMKAVNAQKWNNLAYVELGNKIDALCSDRVLQTGRKECEIAVDFRKCKTELIQQFGGPPKPGST

>AlucOBP17

GELPEEMREMAQGLHDSCVGETGVDNGLIAPCAKGSFADDPKLKCYFKCVFGNLGVISDDGELDAEAFASILPDNMQALLPTIRGCGSTTGADPCDLAMNFNKCLQKADPVNFMVI

>AlucOBP18

APSANVKEIVQNVSKKCAAETKASPDQAKIVLSKNIPKDDAERCFLQCVYTGVGVIKDGKFSEEGGKKLVALRFHDAKEKELANKLIATCAKEIKAKDGEKCSLGRAVRECFVNHGKQVNFFPSA

>AlucOBP19

GNIKEGYVAKIAEIKDKCLKEHNVDHSVVEDLLKKSIKPEVKAAQCMVACFFEENGMMKDGKIVSEMVKSNNAHQYEDPADVEKANEASDMCDGEVSTDGKDKCLLAADYALCWVKRTEEAGLPQIDFANSS

>AlucOBP20

APPADEPAECKPMKEKEEEISKCCKLEPVTVKEQAAFVDCMKLVKDTDKKGPPKPEGFECLDDCILSKTGSLGSDKKIDPAKINAAAKTTYTGDWAEPGAKMVEKCLAQVAENKDKTVCSTSGADVYTKCIFRESYINCPEKSWTNSDACKANKERVIKCPKTLPYNAEQHKAETR

>AlucOBP21

MKFFVVSAALVLLVAAAVKANEKKANEKVTEIFNKCKETWPVTDEEIEQVKQKQSIPDSKNVKCILACMLKEAKILRDGEYNKDNAELMADVLYKDEPEHAEKSKQIIEMCSSELGTKTEGDDCEYAYKMSVCASKHAKELGVKTPEF

>AlucOBP22

YQDQLKQTIKDCQGGKEVTDEELEEFTKPLIPKNEEERCIMACVMRTYNIINNGHYDPKIAFGIIKGILKDHPEKLDRIKEVMDHCGEDVPQHMDNECDLAGEIMQCEVKYQKAMGLN

>AlucOBP23

HPGHFDEDPECRPPHSHRHEEKECCKTPNLFSKNKDEMHELVHKCFEEAGIKKPHHGHHGPPPPGDEPPPPPPPPFHSKNNTKFECVEQCFLKNLELIDEEGDLKIDDFKALVGEKYTGDWASVGSAALEKCLEKTKTEEKESSKCKAGSKHVLLCIARESFINCPASDWTESEVCSDAKERVVKCPDIPPPMNH

>AlucOBP24

YQEQLKETIKQCQDGREVTDDEVEEFTKPLVPKNQEERCLVACVFKEYKVIIDGHFDPVNALNVAKMVYKDYPEKWKRIKDVIDHCGEDIPTHNDNECDLAGDIMNCEVKYLNSMPKGVSLELLAGSIAATAEP

>AlucOBP25

QMDDDPECRPPPPPNKEGSCCTVPRLLDNADKPEVIKKCHDEAGMKRPSGPPGSGTPPTAEEMAAHKSAHECADECIFKSSNLLKSDGELDQDAIKATTTKMFTGDWSTIASTAVEKCLATAKSEVGASAKCKSGAHQMVKCFARTMFLNCPASSWTESTECAAAKTRLTKCPNAMPPPPHHSRH

>AlucOBP26

NTKELSPSEALKQKVKVQCQQEVKATPEQLKIYDNFKDVPKDDVENCLMECMYTKTGGIGADGKYSVEGFKKLVDMKYKGEENTKARKIAADCEAKAAPKEGEKCSMGRAIRECLAAATKENEFFTI

>AlucOBP27

KELTEEQRTQLFEDLKQCKNSTDLSDDEFETIIAKKELPTSEAGKCFTKCLMEKLDIIEDAEGGKKKISVITMQASLEENMEKEDDIAKGKDIIQKCGDTVEPEDSCAYAYNISKCIYDRMKEAGISQ

>AlucOBP28

MIIEIICVLTVGISPHFIEGQELPPPGGVGNKTAVFKESFIRTAKYCSSIHETSTVAVLAILMSEESDDQNGKCFLNCMLQRYQLMSKQGAYNKDKFKPFLDYIPESRFLQSIKGNLKTCITERDPAPCEKAYKFIKCFYTRARNKDEFGKIQRK

>AlucOBP29

QQEDCKTAPAGWPRRPPQCCDLPFPLEGMKKEFGSCIRQIGNRQSSAVPTAQAVRDARLCIEECVYKGLGFMEEHNLNKDQILQQLTKGVADKKDWTKPMEDAVKSCHETITKRETPQEGTCKDSAHEFTHCVMRQLFLSCPASEWNNNDECNLVKSRMQACPNIPPPPPPPPQGFRGQGPPPPQ

>AlucOBP30

TPTTPTPATSRRVTVAPEDLEQAKSLRKFCTAKTGFTGITTTETSKGKDQARTATTPRPKTQLEKCYLECLYTGLQLTKDGKFNEPGARALANKRYKNAPEELRKVNSIIDFCITEVVVRDIEEMCALGRLIKECFSKYGAKNFPEL

>AlucOBP31

QIDEDPECRPSGPPGKEPECCTIPMKLFGDEVQEAVVKNCFDEAGMKRPSGPHGGGSPPTAEEMAAHISAHECADECVFKSGNFIKSDGGLDEDAIKAVIAKLFTGDWAPIATAAVNKCLASAKSGVSASAKCKSGAYQLSKCFQRELFLGCPASLWTESTDCSAIKARITKCPNAKVPIGHHHKH

>AlucOBP32

EVLTDGDCPKTMPKEMKPLYKCCVVEMDSNKTISDDQKAAVDSCVNTSKSDSDANKHDCMIECIFIKLGYMGEDKTINVDYVLKEMNSLLPEDFHEQTSKSLATCMGKKFSSTECPSEIDGVMACFSTMVLMNCPAKHWTDDEECKATRKFFQKCGDSIGYRYD

>AlucOBP33

GLPFQNEMAVMQCKVKFDVTAEDIQLLKDSKLPSSHSGKCMMACILKKMKVMTKRGQFDLRNVQKWLRNKYQGDQANLAKGNYVAEACANTLPTLGIQDECEMAAEIMTCVRTKSKLVKKTLNGELPKEVSP

>AlucOBP34

AQKSKQPSKAKTKESQVVAARPKDARAAACVTQIGPDEEEEASFYRKEIPETDKGKCLLACYLESKGVLSGGKFSSSGAAKIAARAYPNNAAKTGNVKHILSHCGTIAARETEQCQLAYRLAECTTTLADKFKL

>AlucOBP35

GFMEALIECKQQHHVSKEEAMTGESEEVKCFSECVLKKSGMMSDNNEFDEEKIQAEGARMIKNDEQKNREFEGAAKACIEKVNGENPSEKCAKGHALFKCMKEAMPMSKMRG

>AlucOBP36

VSKEYHDKAIAAKNTCAKLHNVDDETIMKFWKAHQLPEKEPETCIIICYMKEMKLVVDGKVDADAWKASNKEKWDDEKHVAAADEIVDKCSAEVPPTENECEWGLALTKCALKHGKEAGIPPPDMEHPKRR

>AlucOBP37

VINKDYLEKVVTAKDKCLKEFNVDDSVVEDFIVRYNKPQSESGKCMVACYMEERGMMKDGKTITEQVMLDNQEKWIAATHVNMGKEVIDTCDKEVPNEKNDKCDLAVDYMMCLVKRGDEAGLPKMDVAQLKH

>AlucOBP38

KELTDEQKEQIFAEIKNCMESTKLTDEEFESIMAKKELPTSKEGKCFTKCLMEKMEYLEEGGKINVIAVQAGLEENMEKESEITKAKEIIQQCADTVPPEDSCEYAYGISQCMYTKMKEAGISGGP

>AgosOBP10

RPQPDEPDDIKKTLYNACSEKFPLTEEIKNNVKNSMVIDDQNFKCFLRCCFDEMSLIDEDGIIDGESLAAMAVDKIKPVAEKIVHDCLPAGKQEKQDGCEAAFKFFSCGMKLNPLTIELLPLQ

>AgosOBP9

ADDADTADKELMSKLITVAFKCFKDADWGTCGEMITTKYDITQAKYKQCTCHMACAGEDLGLINSNGQPEPAKFLEYVKRINNSVIKSQLQHIYDKCQNVKGTEKCDLAEQFAICAFKESPEMKERVTKLIEMLVKMKPKSK

>AgosOBP8

ENNQQNSNDRSASIFQSCISETKLSGDALKGFRSMSIPKTQAEKCMMGCLMRKVNVINKGKFSVEEATKVAQKYYGTNESMMKKAKDLIDVCAKKAQSTTEECALAGIVTTCIVEEAQKAGLTGGPGSRSKRTVSPKFRHSIV

>AgosOBP7

YLSEEAIKKTQKMLKNVCSKKHSVEEEVFTDIKKGIFPENNNNIKCYFACNFKTMQMVNQKGILDKKMFKDKMTMLAPPNVLAILLPPIEQCIGNDKDTEICQSSYNFIKCAHRVDPKSLEFLPL

>AgosOBP6

VGFERTWILRQKRMTNDNECRALFPSPEKKLPTCCQMPNILPGLDNAWEVCFEKFKQFKDKHATKEYKEMVHENEPPCLFQCVFMQSGLTTSDGKVNEDAVIKKMAEGMDNDEKWKSIWRNTFNKCLNDVKQEDKEQIKVMNTPTGRLMKCFLRDLYMNCPKNVWVENSECSNLKDLVEKCPKLPPPVFQSPPKLI

>AgosOBP5

EAGHQRRGKELLDTEDSDFFRCKQASRKSCCGPENAMKRFGDKDKVAADECYAQVAEKFATVTATTPKQDLFSGEAVKITKKKQFCLHECIGKKNKLLTEDGSLNKTFIADYAMKSVFKEQWQKQIGQKALDKCLEETYIPWPAEETENKCNPVYVQFQHCLWLEYESNCPDNKIKLTKKCEKTRNRYRMQKSPSNQ

>AgosOBP4

QKQELSGKCRAPDKAPLNLEIIINICQEEIKSALLQEALDILNDGTLEQNTPSYSRSKRDADEDLSNEERRVAGCLLQCVYKKVKAVDETGFPVVDGLMKLYNEGVQDRNYYMATLSAVRHCISIAQQLKQQQPSKSFDDGQTCDLAYEMFECVSEKIEENCGVENKLNNLSQRQV

>AgosOBP3

RFTTEQIDHYGKACNATEDDLVIVKSYKVPTSDTGKCLMKCMISKLGLLNDDGSYNKTGMEAGLKKYWSEWSTDTIESINNKCYEEALLVSKDIIATCNYAYVVMACLNKQLKLDNST

>AgosOBP2

SDPCNISTCYKSGTTKPPTTVTPTRLPVQSSSTPTSHQQTTYAKDHVHSSTATKSGVNTTATTTSGASVNGTERTTVVKSSSGVAGNVTTPKPTMTDGHVALKQKLNTIAVKCKDELHAPQEIMALVSNTVVPQNEQQRCYLECVYKNLNLIKNNKFSVDDGKAMAKIRFANQPEEHKKAVTIIETCEKEAIIDPKTTEKCAAGRVIRNCFVKNGEKINFFPKA

>RpedOBP1

MAPTASENQQCAKQSSGEKCCTILSSIPPMAFDLLFSKDVKNPTILKCAEDRGSPKDLVHECTGECSFQALGLIKPDKTINNDMILMMAKQFFGRDKYWKDIAITSVKQCLISAPKEVANRSVCKSGVAQFILCIKRDMFLTKNAMKREIMWSNAVRLNQFLFSRETNAE

>RpedOBP2

MGPPSSGSSGIIDWPIWLVLIDGYLSNRDSRHEGNDRNNHRNNYHQTQGKKPSSYDKEENDEDADYDYNSENQNSNRRGGNDWKSQKGQYSQNNEYYANQNHGNRESHNQPQGTANNSKNIGSPLEGVEPCIIHCIFKQKKMLNNRNYIDKSNTIRVLTHKVYNAELKEFIEDSINECLDILHNGNSDGKCEDSKHFALCLEEKGKKNCEDWDAENHRNSNKNSNTNKVYYMGNERLDSYNEKNQNRPTNGGSYGSRGERGDQNKYSNDREYSDDRKNNGYNYNQNNRESQKGKMKNEQERDENSRNRGHINQNDENNEQRKLQNYYQNRG

>RpedOBP3

AKSQKKAVCEAPTVAPQKLEKVINQCQDEIKYALLQEALSALGDAVINRRTKRETFTGEERRIAGCLLQCVYRKMKAVDESGLPTGPALVEIFTEGVKDRNYYLATIQSVQQCLAKEIQQRKANPIMAKAEGYTCDVAYDMFMCVSEQIELLCGTTP

>RpedOBP4

DGTTEAPTTQFASSTVSGATAKTDDVRHKIKEQVTALTESCKNTAKITADQAKIVSNQLIPKTESERCFLECIYQGLNITKDGKFNEAGARGLAQLRFATSPDDLAKANGMIDACIKEIVVKDASEKCVMGRLIRECFVKHGAKINFFPKP

>RpedOBP5

YGPQGGPPWDPNLPAECMPRRGQFGEQVDVCCPLPGFSPTQGFQNDGIHEKMSQCRQTLGNLPGGQGSHGFRGDRECFDECLFSLDGLLSSDLKLIGEEVRKKMLEVHGGDPTWSPILEQATVYCLQTAPAEVQATSQCRSGAMQFKKCFMRQLFLNCPSSYWNNSESCLAEKALLERCPNLKPRRPRFN

>RpedOBP6

YPNDPKPAPPDMDEPEECKPKPPEPGKEREDCCTMPECPQNEEKMKIMKECSDMLRDQMPPPPKADGHPPPPPPTGAGSPPPPPPPPGSAHPPPPPPGSEHPPPPPPPGAKHPPPPPPPGAKHPPPPPPHHHHHHHGECMEECLFNKTGFLTSDMKLNGDEMVKKIVEEYGKEEAWKSVVEQAVKTCISSSAEGVKEDAKCKSGSHQFTMCFERQLFLNCPSGVWKNTDSCNAEKTLLTKCKNRMPPPPPPPMPKNKTE

>RpedOBP7

MSYVSSIQSAMGILSDNTYVLGILIVVAYKVLAEDSDIPKECRNVLSLETEEKCCPIPFGQMSEKELAYMTECTAMPRKGLPSTDAPEESSSEESSEEENINIEDEECIDECFFNKTGLLTLEGKLNEEVVRKDFLEDFNSEPWKPVAEEAITKCLVKAPSAVKEGAKCRSGSYQILLCVLSEIYKNCPPGVWLDSSACKKDKALTEKCVEYVIR

>RpedOBP8

DDAADECRPKPPEPGKEREVCCKIPDLIKKKEGVEMHTALHECMESLKANHPDLFPEGLPGPPAPGARPPHPRGPPPIKVIECIQECFFNNSGLMTADMKPNSEAILGLAENFVETDKDWKPISDEAIKKCTASPPKVKDDAVCKSGAHQISLCIVREAYLHCPPAHWTESEACKAEKALMEKCEDMPPPFFPYNKHKKHH

>RpedOBP9

MWWGVMLYKGSYRQSASVMARMYSLPLSFALFFLGVAGVFALDIDFHSDLVPELSPSTAVQHRTRFTRGTVGNEAEIKKDIEKGEWWKRRCCGNGTSYNPEFAKKKRQFIDECTPETLSKSTHVISADPFLESCQDLKEKNRRSVCNVDCVYRKYGIVDASGNINSEKLKELSEEFVTSDWLRQIRAKAIEKCAKWKGEDIETTEPKCNQNAYNLKHCMWKEVVITCPEEHRYKSDYCDNVRKSLIPK

>RpedOBP10

EEDTNSVEQLCEKETNVPQNDIENYKEMRLPQTREGKCYGACIMKTVEMINSRGKIDERNIKEYAGTTIKNYDQLDNVEHIVRRCVKKANHQRDECDTAYQFMDCLMRRKMSKTSVQVTLQIPEPNINLPPVTLVLFQLGG

>RpedOBP11

GKTDMAEKIIRTMRKCSRQHHLVLKDIEGAIKHGDLPQSEDGKCFVTCTLKEFGLVSSDNKFDPEKSFALNALRFKNPDDVQKANDVTKKCAEELTVEFENPCDFGIAVANCGVKHSREAGIINYNLW

>RpedOBP12

RPEGPDARKQFLRAEKAILEKCSKETKAPKGFVDDIFVREVVPSSKEGKCFTKCALQNLKWMKSDGSLDAVAIAKLADKAYFNKEQNAKAAGIWKKCYAEQPKTGNDHCELAYKALNCYFTGAKAAGLPRHSQGA

>RpedOBP13

QEVDCSKPPDGWPKRPPQCCDLPFPLEDMKRSFVGCIRQIGRPTSAVPTPKSVRDTRLVSVLCLEECVYRGVGFIRDGDNDLQKDAIQTKLKGLLQEIPAWEKAISTATETCFDEDKSAEESSEESSCSAAPHDFTHCLLRNLFLHCPADLWNKSDECELVKNRMKECPELPPPPPPPPQRFQRPPGPPPF

>RpedOBP14

ASTSQAGVAEKVKALAKKCAADVKLPEDKAKLAFNQEIPTTDAERCFLECCYKGLNVVQEDKFSLEGAMKLVHARFQKPEEVSTATKLVETCNKEVSANAAEKCSIGKSVRECFVKNGKEVNFFPSAL

>RpedOBP15

LELTEETKGYLEECQKVHHIQPEEIVAAVKDKKLPESENGRCFLECMMEKFGVLHDGAFDTEKANAIAATKLQNKPDVLEKATKIIETCNTEVKNPGGKCEYGVELAKCAMKYAKEMGIQDAPIGN

>RpedOBP16

ETPVLKTPAEIKLVFETCGKEVGYEGTMSSARSEPTKESKCFVKCVMEKKGVLKGDGTIDSELAKAYLSVMPQFKNNPEIKEKALKAVDDCAAEVKINMDDLCESVYEMFVCRHRKTVA

>RpedOBP17

QDKMNEKIQKAFNNCKEKHNPPEVDLEMMRKSDLEYKYSREAKCLIACMLEEGKIMKDGKYNKENALIMSDVLHKDDAEDAAKAREVVETCVKDHPDGGTDKCEFAYEMAICGGQEAKKLGMKKTPDFFNP

>RpedOBP18

EPEEDIRKLETEILDVCKEKTKASQDAVNQLLKSGDLDESPEGKCLIKCGLQTFGTMTEDGAFNNIRARDYMKGAYPENVDEAAEIWENCQAHAKKTDDKCDYAYKVIKCFFAGIKNQNIKKAW

>RpedOBP19

QEPKGTQFKKRFLRYSSHCAQQHSTTLGTVPALNSQAPTLVDIMGLLAGEETSSSSGKCYILCMLAQYKIINRDGGYRRETFKEFLNSMPESRFKKSLEASSQDCYKQDNQEECEKAYRFARCFYTSAISSKKYP

>RpedOBP20

KDEIERGILEECNKKFNLPPSVIDRYLETDELDGSRDGKCLWKCYLIGLKVMNADGSMSEEGSVYFALHAWKNPDDQQKELKVWHYCQKQAENPDTCEFSHRLLTCYFIEAEKVKIPRRDFET

>RpedOBP21

AMTQAQMKQAMKTVRNMCLPKSGASKEALEKMSNGEFDDDDRKLKCYLGCVMGMMQAVKKGKIDLKMVRSQVSKMLTPEFGSKLLQTFESCQGAEGADNCELAYNFAKCIYETDKESFIVP

>RpedOBP22

ALTAGEWDYLWEKAEKKCEHLMKFGPNDKKSLLTEIDDIPRRAKCFVSCFFDEVGLTSGTEVNHELYMKWLDEELQLAKNKDEIVESVKNCIDGIEKNDKCQTAYQLYECFGKRYFSLN

>RpedOBP23

MQCLAKYKVPSRDVENIKNRKMPQTKQGKCFMACILKRLNVINESGKFEVSTVKSWMADKYKDNVQKLNKAYAHADMCAELLPSKSGTDECEYAAKILECSRQRKRIRN

>RpedOBP24

RGGFLASRPSRGPLSETVGEDIQACKEEHNVTDGDLEKMRNKDIPVTVEGKCFLECVMNKVDLMSGDRLNPEGAKGMIEEIDNISKEDKAKLGEVIDECDKEIEYKEDEDCENAIIVSKCISQKAAQKGVQPPEGV

>RpedOBP25

ATKPEFLKKVSEHIELCSKENNVDKVEVTKHLTERILPEKQEVKCTITCYLEKAGYLKEGKLDWETAKETNQHKYDDEESVKKANEVADICSKSVSIEGLEKCDISNAVLKCLFEESEKLQLAHPDFLGKLTSA

>RpedOBP26

MRNTNTSTSEKICDYLMYNKFCVYACGTPTQQLLNCYLQRAGYLSQGVINWSALKFSNKFRYDDEISIGKADKVVEGCQNEIESSVVPFDDIGKALLECWFKHSEEAQLQLPEFLQRPPNS

>RpedOBP27

SDRDEYFKKATRNLSYCLSSYGVKHDGLLAMLRREQPDKTRLFITRISFVCYLRRSGYLHGGKFNWDLLKEDNKFRYGDEESLKKADQVVSACKLKYGRNPILTQHLIGCVHEEAEKVQLTDAYYLLAPIPI

>RpedOBP28

NFDSKYYRIMSASVNYCGKEDPTVKGGIERLVEILSKRILPEKPVEKRMLECYLVSVGYLTPPSKPINWDFVKAYNDQKYKYTHLEEEWKKSNLVIEACRGKVPVRDESTVINVFKCLYEESDKLQIELPELSPDIIEMATRRIRVKVAYLKHIAESINECAGHFPKRRDWKQALVTMVTQHILPKNRDEELLLECYLDKSHYLKYTFLHPMGIDWNKIKRYNKVKYNDKIMREESSKVIDACRNKVKFEDSFKSTKIYECLYEEAAKLQIELPEFSAKILNQIMKPGEMEK

>RpedOBP29

ESKAETFRDILEKCNEEIKLPQSVIDKIMNSFELDDSKDGKCLLKCSLEALGVMKDDGSIDEEASKKYAAVHFSNIEQREKNLEHWADCQKKVKKGDCHCEYAYNLLACQAEGPHKELLFIPDPKH

>RpedOBP30

ESKADTFRDMLEKCKEDMKLPQSVLDRILNDFELEVSKDGKCLLKCGLEAIGVMKDDGSIDEEASKKYAADFFSNIELREKSLLHWADCQKKVAKGDDTCEYAYRLVVCQREGRTKELIFNAEPKH

>RpedOBP31

MNLFSAPENATKEAKCFLKCFLENKQVLKQDGSVDADVGKAYINASENLDAERKAKISKAIEECSVEVKPNPDDLCESAFTFVMCKMKKMNTPA

>RpedOBP32

MRIGTTFINLVKKVKKDSKVVFRLCFISELQTGDDQNQARTHLPAAMDTLLPAILLAAIAVVLCDSPPPKTKEEVKAIMDKCRQEHGFQGSLKDITPENATKEAKCYLKCFFENKEVLRKDGTWDVEAAKAYLNAAENLDAERKAKLIKATEECSKEVKPNPDDLCESVFEFIMCKKKKVNPPA

>RpedOBP33

YEMKNMEEIVEKCQKEIGFEGTLTTIGTDAAPKEAKCFLKCVMEKKEVLRKDGTIDKDLAKFYLENMKIPAARKEKLLNVIDACTAETKPDPSDLCGTAYKYFQCKIKKADGA

>RpedOBP34

MYAKEVALFVVLASTMNFAYGLDIRGLLKTRLNVEECRRQNNIEMDQIMSAVRDRKLPEKEGGKCFLECMMQKAGVLENGKINARKAKNINEKNFGDKPDIKAKADEVIDRCSTVENPGGKCQYGLEITKCVLDNAKELGIPEPSFIHS

>RpedOBP35

FEVQLSDKAKALVEVCRKENNLEMDQIKDAVEALKVPETEAGKCFVECMYEKAELVKDGQINVERIKALNAKKLADHPEMLEKVNQVTDKCSKVQNPGGKCAFGVEILKCALSTGKELGIPAPNFIY

>RpedOBP36

VELSDRAKQLLEECRKENNLDAEKLKEAFKNRKIGETEGGKCFLECSMKKFGIIQDGKYNIERAKSINANRIKDNPELLAKANQVVEKCSKVQNPGGNCGFGIELAKCYAEAGKEAGITDQIFKM

>RpedOBP37

MSDEMKEMAKMVHDQCLEESGASEDAIENTKKGVFPEDQKLKCYLKCIYSNMGAISDDGALDADAFSSILPDDMQYMQPIIDACKGVTGSDGCETAINFNKCMYKTDPGKYLII

>RpedOBP38

GPVDEAKQMLQTELLTYEKCGKENNIAEAELERIISHYEIADTRNAKCLASCHLRGMKMMSEDGVVNWDRVDEIHQIEYVKPEDDAKALEVTKACRNSVKGTADHCDTAYLALKCFLDNAKKANLHLLGTEDLQ

>RpedOBP39

SDVDPKMKANLEECAKKNNLGMDDMKSMIMNGTLPYTEAGKCYMDCMLEKIGVLEGGKLNVEKAKAINAITFKDNPEVREKTDLLAEKCSKEVVSGNKCEFGTELTKCSKKYAKEMNIPEYSFS

>RpedOBP40

SDVDPKMKANLEECAKKNNLGMDDMKGMIMNGTLPYTEAGKCYMDCMLEKIGVLEGGKLNVEKAKAINAITFKDNPEVREKTDLLADKCSKEVVSGNKCEFGTELTKCSKKYAKEMNIPEYSFS

>RpedOBP41

TDDMKEGAKALVEECAKKNNLEMDKVMEIIMSKKIPESEDGKCFMDCMLEKMGMVENGKLNVEKAKALNAIKLKDKPDVREKADEVVEKCSKEVVNSGKKCEYGVELTKCAIKYSKEMNIPEPTLT

>RpedOBP42

GELDCWDYKSFVMKTKDCCQHNDDDDDDEEDLKEGISEHALHCVKKYAEGSPVLSREAVLDKIACITECAMNGTKALTADMKLNSELFIHYYDEKDDVESWKTLWKTSVDTCLEKTAKRMTENMKCKSGLMYFLPCLWEETTANCPPELLDTKREECTRVKYYIDKCSIFKTL

>RpedOBP43

ESDMLDKLQEYRKQCMQETGYTDSMAAAKLEDMPQQAKCYVACLAQKEGELTKDGLNVERVKENMHKLFKDDSKVEKIIQVMEVCAKEVKVIEDNLCETAYQYLLCEKKMSDNPV

>RpedOBP44

DKSVMEEVTRLSEECQKESGFSIASSKNITSIDDVPESGKCFLACMAQKTGQLTKDGFNFDKIKENIIKLQDVYKNVDRPINITPKPKSAEEVLKLTESCAKDLKVTEDNLCEVAHQLMVCYFKNGS

>RpedOBP45

EKSVLEEMGKYAEECQKETGFSMASSKNIKSVEDIPEQGKCFVACMAQKTGQLTKEGFNFEKIKENLTKLKEALKSLDKSFNITSNARSTEDELKIMEYCAKGLKVTEDTLCEVTHEFMVCYFKKLESSKA

>RpedOBP46

EKSVLEEMMKYAEECQKESGFSMASSKNIKSVEDIPEQGKCFVACMAQKTGQLTKEGFNLEKIKENLTKLKEALKGLDKSFNITSAPRSTEDELKIMENCAKDIKVTEDNLCDTAHQFIVCYFKKLEPSKA

>RpedOBP47

EDDFLAEFQRVTEECDKETGFSKVPPPLVNSTEPYPEEFKCFFACISQKRGVLSKDGINWEKAFEDEKQLAQKQAEKYKLSEVKLPGEKELKILQDCARELKVIEDNLCETHHQFMFCYDKQMRP

>RpedOBP48

ENESFLKKEVTEECAKETGYKGTIFEAVSDSPSKESKCFVKCVMETNLVLKKDGSIDTDAAKSILSTDRKVKDNEELKSKVYTIIDQCAAQVKPNAVDLCENAHQYFSCEMNKVMPLLN

>RpedOBP49

MISFNEVHSNGSLKASPEIIEECKKESDFSDDIYSIKSDETRKNAKCFLACVMQKKGVMNKEGIIDKDKAKEIVLKEYPQLKEKLFAVIEECSKEDDSSKKDACEAAFKYVMCRRSKLGITPKKD

>AlinCSP6

ADKYTDKYDNIDIDEILTNERLYKKYFDCIQGTGKCTPDGIELKEKIPEALKTECAKCNEKQKAGVEKVMRYLITKKPEDFKILEDKFDPEGVYRKKYEAQRKLVEEGKPVEY

>AlinCSP5

YTTHYDYIDVDQVLNNTRLYTKYVECLLGQGKCTPEARELRDKLPEALQTNCARCSERQASESHRVIRFLIQNRQEDFKLLEAKYDPSGLYFKRFEEETKRNVSLS

>AlinCSP4

EMTEEEFYTKVFEEVDPDFILDNERILTSYLKCFYNEIECNAHAEVVKKSIPDVLATVCGRCSDKQKSIFKYSLNKFIPAHPKDWEKILSIYDPSGEAWPKVKAFIES

>AlinCSP1

AATYTSKYDNIDLDEILSNTRLYKKYFDCLANKGKCTPDGKELKESLPDALKTNCAKCTKKQQEGTDKVFRHVLKNKPNDYKVLESIYDPPGIYRKKYEAEAEKRGIKLPGSH

>AlinCSP2

AEVYTSKYDNIDVDKILSNDRILTRYIKCLMEEGNCTNEGKELKKTLPDALASGCTKCSEKQKAQTEKVLRHLSKNRPRDWALLKTKYDPKGEYSKKYEKEAKALTA

>AlinCSP3

AEQYTDKYDNIDIDEILNNDRMYKNYFHCVMGNGKCTPDGLELKAKIPEALQTECAKCTDKQKKEVEKVLRFIINQKKDDYKLLEEKFDPEGVYRKKYEAQKKLVEEGKPIEY

>AlinCSP11

KFFFSGLLLVCMASVSLCADEYTDKYDSVDLDEILNNQRLYQKYIDCVMGKGKCTPDGALLKEKIPEAS

>AlinCSP10

MRSNFINESIPDVLATVCGRCSDKQKSIFKYSLNKFIPAHPKDWEKILSIYDPSGEAWPKVKAFIES

>AlinCSP9

LAVVTREMREREFFRQLEVINVDSILINQRLIDKYIKCLLKTGKCDPIMKDLRIALPLILGHLCEARCSEK

>AlinCSP8

ADKYTDKYDNIDIDEILNNERLYKKYFDCILGNGKCTPDGTELKETIPDALKTACAKCNDKQKAGVEKVLRHLLTKKAEDYKILEAKFDPEGVYRKKYEAQKKLAEEGKPIAL

>AlinCSP7

ASTYTDKYDNIDLDEILTNERLYKKYFDCIQGKGKCTPDGTELKEAIPDALKTECAKCNAKQKAGVEKVLRHLLTKKAEDYKILEDKFDPEGVYRKKYEAQKKLADEGKPIVL

>AlucCSP8

TKYDNIDLDEILSNQRLYKKYYDCLANKGKCTPDGKELKEALPDALKTNCSKCSKKQQEGTDKVLRYVLKNKPNDYKVLENIYDPSGNYRKRYEDEASKRGIKLPGSH

>AlucCSP7

AELYTDKYDNIDIDEILNNDRMYKNYFNCVMGNGKCTPDGLELKAKIPEALQTECAKCSDKQKKGAEKVLRFIINQKKDDYKLLEEKFDPEGVYRKKYEAQKKLAEEGKPIEY

>AlucCSP6

SELYTDKYDNIDVDEILGNQRLYQKYFDCIQGKGKCTPDGAELKKNIPEALQTDCAKCSEKQKAGVEKVLRHLINEKPEDYKVLEEQFDPEGVYRKKYEHLKKKVEEGKPIEY

>AlucCSP5

AELYTDKYDNIDVDEILGNQRLYQKYFDCIQGKGKCTPDGAELKKNIPEALQTDCAKCSEKQKAGVEKVLRHLINEKPEDYKVLEEQFDPEGVYRKKYEHLKKKVEEGKPV

>AlucCSP4

ADQYTTKYDNIDLDDILKNQRLYKKYFECLTNKGKCTPDGKELKEHLPDALKTGCSKCSEKQRAGSEKVIKHLLKNKPSDYAILEKIYDPQGSYKKKYEAEAKKLGINV

>AlucCSP3

AEVYTSKYDNIDVDKILSNDRILTQYIKCLMEEGNCTNEGKELKKTLPDALASGCTKCSEKQKAQTEKVLRHLSKNRPRDWNRLKNKYDPKGEYSKKYEKEAKAISA

>AlucCSP2

ADKYTDKYDNIDLDEILGNQRLYQKYFDCIQGKGKCTPDGAELKETIPEALKTECAKCSDKQKAGVEKVLRHLIREKPDDYKVLEDQFDPEGVYRKKYEDLKKKVEEGKPIEY

>AlucCSP1

AATYTSKYDNIDLDEILSNTRLYKKYFDCLANKGKCTPDGKELKESLPDALKTNCAKCTKKQQEGTDKVLRHVLKNKPNDYKVLESIYDPTGIYRKKYEIEAEKRGIKLPGSH

>AgosCSP10

MINTRPRKLVRCIRGVSISVAKGDDAVNAENKDDDSHLVNREEIQRYMSMMEKINIDQMLNNTRLMSNNVKCFLNEGPCTAHLREMKKMVPMLVKDSCSSCTKEQKIMMKKAMDAVKARRPNDYEKLSKFFDPEGKYEKKFLENLNESK

>AgosCSP9

TFTRSTKFDDRTGIDIHLVKRDTDDVNDDENSVESDEGFFYRFTHFFQDSSDKEDDDDDEKKPDFITTFDIFKLLDEEYAMQQFYCVINEDPCDEVGMRLKATIPEEINRNCERCTSTERNNIRRILNYVKKHYPQFWKRVEPIYKKKI

>AgosCSP7

RPEDVKVENKPAVIKSETLAAPLPTNIVKRATDTIQLDSSLPNVSEDVLDKALSDRRFVQRQLKCATGEGPCDPIGRKIKAHAPLVLRGMCVKCSQSEIKQIQRVMSHIQKNYPKEYTKMLKQYQSGF

>AgosCSP8

ADGGIITPQQQQQQTMMFTAPTGYYVSTYDHIDVGRLLRNNKVVSGYVKCFVNEGPCTPDGKLVKAYLLPEIIRTVCGKCTPRQKDMARMVLKHIYTYRQADFEKIMQIYDTDGKRNEILAFMNH

>AgosCSP6

APAKYTTKYDNVNIDEILNNDRLVASYFKCLMETGKCTPEGEEIKRWLPEAIENKCEDCSEKQKLGSEKIIKFLFEKKNDMWKQLEAKYDPQGTYRQRYAEEAKKLNINV

>AgosCSP2

AEEKYTTKFDNFDVDKVLNNNRILTSYIKCLLDEGNCTNEGRELKRVLPDALKTDCSKCTDVQKDRSEKVIKFLIKNRSTDFDRLTAKYDPTGEYKKNLEKFEKERASAKPLKA

>AgosCSP4

APQKDAVAASGPAYTTKYDHIDVDQVLASKRLVNSYVQCLLDKKPCTPEGAELRKILPDALKTQCAKCNATQKNAALKVVDRLQKDYDAEWKQLLDKWDPKREHFQKFQQFLAEEKKKGFTKF

>AgosCSP5

SPAGTATAAAVSADDEIKDFPAYMKRFDKLNVEQVLNNDRVLASHLKCFLNEGPCVQQSRDLKRVIPVIANNGCNGCTERQMTTIKKSLNFLRTKKPTEWARLVKIYDPSGTKLNKFLDA

>AgosCSP1

MNILTIFCYVTVMCDTQVKPAVSAQRLQSVNQNVTPTNDGRKTIRETSSYPTRYDYIDIEAVMNNERIIKILFNCVMSRGPCTREGLELKRIVPDAIQTECAKCNERQRKQAGKVLAHLLQYKPEYWKMLVQKFDPNNVYLRKYMADNDDDEKLSLQKLSNDTTKKKRNI

>RpedCSP1

EHRYTEQYDTIDVESVLHNHRLLDNYMLCLRKEHGAKCTPAGRELKANLHDAMESDCKKCTEKQRSNVDKVLEYLKTHHPNYLQDIFNIYDPQFTYRKKHHIKV

>RpedCSP2

MMSSPGEVLRALRKEYLGSSKVNGSFMDVLLVAFIEGGMCADVTVTDEALEAALKDKRYLARQLKCALGEGACDPVGRKLKSYAPLVLRGNCPRCTPQEVRQIQQVLSHIQRNYPKEWNKMLKQYAGM

>RpedCSP3

ATTSYTTKYDNINLDEILNNERLYRKYFECLKGAGKCTPDGKELKELLPDALETDCSKCSEKQKAGSEKVLRFLLEKKPDDYAELEKIYDPQGTYKKKYQEEFEKQGFKL

>RpedCSP4

EMSQDEKEVYERIFNDADVDIIVNNDRILDMYLRCFFNEGPCTTLGKEIKGKISEVFSDVCGRCTLHQKRVWRHAMDIFIPKRPKDWERILSIYDPDGSYWPGIKAFLEGPAP

>RpedCSP5

EEEEDDIYKKIFDDVDVDSILNNDRILDTYLKCFFNTGPCSNLAETMKGKIPEVFSTVCGRCTDKQKQILRHCLHVFIPKRPDDWKKILEIYDPEGKYWPGIKAFMDEDPNA

>RpedCSP6

YTTKYDNIDLDEILKNDRLLNKYLDCLMERGKCTEDGKELKEALPDALKTDCSKCSEKQKGGTKKVINYLVEHKRANFDELAAKYDPEGVYKAKYNEEAKKNGNKLIE

>RpedCSP7

YTTKYDNIDLDEILKNDRLLNKYLDCLMERGKCTEDGKELKEALPDALKTDCSKCSEKQKGGTKKVINYLVEHKRANFDELAAKYDPEGVYKAKYNEEAKKNGNKLIE

>RpedCSP8

LPTPGDEKYTDKYDNINLDEILGNERLFGQYLACFKGKGKCSPDAKYVKDVLPEAMADNCSKCSDKQRQNSERVIKFMVEKKRPDFEEIENMYDKDCKWRTTWRDYYAKRGITLVECKH

>RpedCSP9

ASTYTSKYDSINLDEILNNERLYRKYVDCLKGLGRCTPDGRELKEHLPEALENGCSRCTAKQRAGSEKVLKFLIKNRKEDFAELEKIYDPNGIYRKKYREDASRAGLQLE

>RpedCSP10

KPADTYTTKYDNVNLEEILSNDRLYKKYFDCLAGKGKCTPDGKQLKDVLPEALATQCKKCTERQRKGSERVLKFVIEKKPQDWAVLEKIYDPQGTYRQKYKQEAEKRGIKI

>RpedCSP11

SLVYTPPTTTLMPYDSDFDDIDLEEILQDRSKFVPRVNCLLDLSECPPDAAQVKQNLAEALDTGCRRCTEKQRAGALKVMSELSNYHPELMQKLYDRFDPEGLYRTKYKLEFIDAGIKLLQQP

>RpedCSP12

DEGKFTTKYDNVNVDEVLHNQRLLENYLKCLLDEGKCTPDGQELKESIPEALKDKCAKCSEKQKEGTKKVIPFLVKEHPDYFLKLEKKYDPEGIWRKTYEPEAKKLGIELPPKQE

>RpedCSP13

RPQDSATMSEGSEAPKLPVYPNEEDKSAKTQEVTPKSDESQGVSNKSQDQESTKSSSSKSRLSTYTTRYDNVDLDEILNNNRLFRKYYSCIIGEGKCTPDGKELKELLPEALATDCNKCTEKQRAGGEKVLKHIREKRPNEWAILQKMFDPQGVYSSRYNAQGMAKMAMEE

>RpedCSP14

DETYTTKYDNIDLDEILKNDRLLNKYFECVMERAKCTEDGKALKEAIPDALQTDCSKCSEKQKAGTEKVIKHLVEHKRAMFDELTAKYDPEGIFTKKYNEEAKKHGIKLIE

>RpedCSP15

DETYTTKYDNIDLDEILKNDRLLNKYYECVMERAKCTEDGKALKEAIPDALQTDCSKCSEKQKAGTEKVIKHLVEHKRAMFDELTAKYDPEGIFTKKYNEEAKKHGIKLIE

>RpedCSP16

YTTKYDNIDLDEILKNDRLLNKYFECVMERGKCTEDGKALKEAIPDALQTDCSKCTEKQKSGTAKVIKFLVEHKKAMFDELSAKYDPEGIYKKKYNEEAKAKGIKLID

>RpedCSP17

YTTKYDNIDLDEILKNDRLLNKYLDCLMERGKCTEDGKELKEAIPDALNTDCSKCNEKQKSGTTKVIKFLVEHKKAMFDELCSKYDPEGIYKKKYNEEAKEKGIKLLD

>RpedCSP18

YTTKYDNIDLDEILKNDRLLNKYLDCLMERGKCTEDGKELKEAIPDALKTDCSKCNEKQKAGTTKVIKFLVEHKKAMFDELCSKYDPEGIYKKKYNEEAKEKGIKLLD

>RpedCSP19

YTTKYDNIDLDEILKNDRLLNKYLDCLMERGKCTEDGKELKEAIPDALKTDCSKCNEKQKSGTTKVIKFLVEHKKAMFDELCSKYDPEGIYKKKYNEEAKEKGIKLID

>RpedCSP20

KPQQTYSTKYDNIDLDEVLSNERLYKNYFNCLTGEGKCTEDAKLLKEAIPDALQNECSKCSEKQKAGGEKVLKFVIENRPEDFKKLEDIYDPDKIYRKKYNEEAEKHGIKLID

>RpedCSP21

KPQETYSTKYDNIDVDEVLNNERLYKNYFNCITGEGKCTEDAKLLKEAIPDALQNECSKCSEKQKAGGEKVLKFVIDNRPDDFKKLEDIYDPDQIYRKKYNEEAKKHGIKLIPE

>RpedCSP22

APRPAGGGTYTTRYDNIDLDEILRNDRLYRKYFDCMVNKGKCTPDGKELKDNLPDALATGCSKCSEKQKAGTEKVLKHLLAKKPSDYDQLEKIYDPSGVYRKKYQSEAKKHGVNIH

>RpedCSP23

AEVYTSKYDNIDVDKILANDRILTQYIKCLMEEGNCTNEGRELKKTLPDALSSGCAKCSEKQRSQTEKVLRHLSKNRPRDWARLKTKYDPSGEYSKKYEGKFTQEPSS

>RpedCSP24

MVIDVERILTNNRIITKYIKCMLRQGVCPPEARDFRRTIPVILRHLCENCTDRQRNALKLIFTFVKENYPDEWGRLMKLYDPKALRVSKSVIKEKYAEGNESDAGAMEYTHDPDSDIKMPHLISLEPELDQLEVLVQDKSPSKEKSELLDSRLNE

>RpedCSP25

LPANTYSTKYDNVDIDSILKNDRILKKYIDCLMDRGRCTPDGQMLKDVLPDALKTDCQKCSAAQKKMAGKALAYIIQYKRHYWNEILEKYDPEGSFRKKYEYEDD
